# A Novel Dual‐Network Approach for Real‐Time Liveweight Estimation in Precision Livestock Management

**DOI:** 10.1002/advs.202417682

**Published:** 2025-04-26

**Authors:** Ximing Dong, Caiming Zhang, Peiyuan Wang, Dexuan Chen, Gang Jun Tu, Shuhong Zhao, Tao Xiang

**Affiliations:** ^1^ Key Laboratory of Agricultural Animal Genetics Breeding and Reproduction of Ministry of Education & Key Laboratory of Swine Genetics and Breeding of Ministry of Agriculture Huazhong Agricultural University Wuhan 430070 China

**Keywords:** automated livestock monitoring, contour‐based estimation, deep learning techniques, non‐invasive weight measurement, precision agriculture

## Abstract

The increasing demand for automation in livestock farming scenarios highlights the need for effective noncontact measurement methods. The current methods typically require either fixed postures and specific positions of the target animals or high computational demands, making them difficult to implement in practical situations. In this study, a novel dual‐network framework is presented that extracts accurate contour information instead of segmented images from unconstrained pigs and then directly employs this information to obtain precise liveweight estimates. The experimental results demonstrate that the developed framework achieves high accuracy, providing liveweight estimates with an R^2^ value of 0.993. When contour information is used directly to estimate the liveweight, the real‐time performance of the framework can reach 1131.6 FPS. This achievement sets a new benchmark for accuracy and efficiency in non‐contact liveweight estimation. Moreover, the framework holds significant practical value, equipping farmers with a robust and scalable tool for precision livestock management in dynamic, real‐world farming environments. Additionally, the Liveweight and Instance Segmentation Annotation of Pigs dataset is introduced as a comprehensive resource designed to support further advancements and validation in this field.

## Graphical abstract

A novel dual‐network framework is proposed for real‐time, non‐contact liveweight estimation of pigs. By extracting contour information instead of segmented images, the method achieves high accuracy (R^2^ = 0.993) and an exceptional speed of 1131.6 FPS. This approach enhances automation in livestock farming, providing a scalable and efficient solution for precision livestock management.

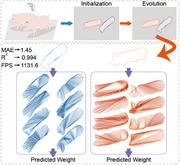

## 1 Introduction

Noncontact measurements play a vital role in smart farming by increasing both the efficiency and accuracy of livestock management processes. By eliminating the need for direct physical interactions, noncontact methods reduce stress and minimize the risk of injury to animals while also decreasing the labor and time required for monitoring and data collection tasks.^[^ ^1^ ^]^ In this context, liveweight estimation is a critical tool for evaluating smart farming performance. Accurate and frequent animal weight monitoring provides valuable insights into growth rates, health statuses, feeding efficiency levels, and the effectiveness of management strategies.^[^ ^2^, ^3^ ^]^ Additionally, implementing precise feeding programs and ensuring that animals reach their target market weights are essential.^[^ ^4^ ^]^ As the demand for automation in pig farming continues to grow,^[^ ^5^ ^]^ particularly given the key role of this industry in global protein and fat production,^[^ ^6^, ^7^ ^]^ noncontact liveweight estimation has become a central focus with respect to the advancement of smart farming technologies.

In recent years, advancements have been made in the non‐contact pig liveweight estimation domain via computer vision and deep learning techniques. The traditional computer vision‐based methods rely heavily on extracting morphological features, such as body length, width, volume, and back area information, to estimate liveweights through regression or machine learning models.^[^ ^8^, ^9^, ^10^, ^11^, ^12^ ^]^ While these methods can be effective in controlled environments, they often require the target pigs to exhibit fixed postures or be located in specific regions for obtaining accurate measurements. This reliance on controlled conditions limits the adaptability and practicality of such methods in dynamic, real‐world farm environments where animals move freely. With the advancement of deep learning, convolutional neural networks (CNNs) have emerged as promising solutions for addressing these limitations. CNNs can estimate liveweights from unconstrained, full‐body pig images,^[^ ^13^, ^14^, ^15^, ^16^ ^]^ offering increased flexibility and adaptability to natural farming conditions. However, this flexibility comes at a cost: CNNs typically require inputs to propagate through multiple convolution layers, increasing the required computational time and the demand for high‐end processing hardware. In resource‐limited environments or in cases where real‐time liveweight estimation is crucial, this type of approach can become inefficient and costly.

Since the positional features and geometric shapes of pigs are implicitly employed by CNNs to estimate their liveweights, this study explores the impacts of these factors on CNN‐based liveweight prediction, revealing their critical role in attaining improved accuracy. Contour attributes, which is represented by the coordinates of points that are uniformly sampled along the contour, inherently consist of both positional and geometric aspects. The coordinates convey positional details, whereas the geometric shapes of pigs are captured through the sampling process implemented along the contour. On the basis of these attributes, positional and geometric characteristics can be effectively leveraged by networks for liveweight estimation. Consequently, it is hypothesized that a more direct and explicit scheme for utilizing this information could lead to further improvements in both accuracy and efficiency.

To address this challenge, we propose a novel dual‐network framework consisting of a contour information extraction network (CIEN) and a liveweight estimation network (LWEN). This framework is designed to optimize both the contour extraction and liveweight prediction processes in a more efficient and scalable manner. By integrating the CIEN for precise contour extraction and the LWEN for accurate liveweight estimation, the proposed approach overcomes the limitations of the existing methods, providing a practical and robust solution for conducting liveweight estimation in real‐world, unconstrained environments.

Furthermore, to address the gap in the publicly available datasets that combine liveweight data with corresponding pig images, we introduce the Liveweight and Instance Segmentation Annotation for Pigs (LISAP) dataset. The LISAP dataset offers a comprehensive resource for developing and validating noncontact liveweight estimation methods.

**Figure 1:**
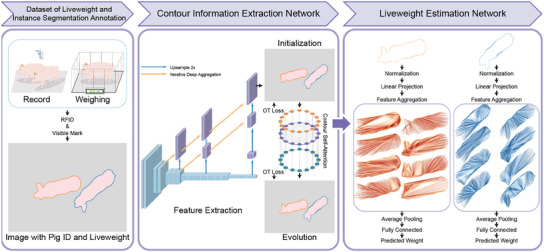
Overview of this study. (left) Flowchart of the LISAP dataset; (middle) architecture of the CIEN; (right) architecture of the LWEN. The figure illustrates the dual‐network framework consisting of the CIEN and LWEN.

**Figure** **1** provides a comprehensive overview of our study. The left part shows the flowchart of the LISAP dataset, detailing the data acquisition and annotation processes. The middle part presents the CIEN, which accurately extracts contour information from pig images. The right part depicts the LWEN, which is designed to convert the refined contour data into precise liveweight estimates. This dual‐network framework integrates the CIEN and LWEN to effectively address the challenges of noncontact liveweight estimation, combining detailed contour extraction with efficient liveweight prediction to increase both the accuracy and real‐time performance of the framework. The diagram demonstrates how the outputs of the CIEN serve as inputs for the LWEN, underscoring the sequential and complementary nature of the networks included within the proposed framework.

## 2 Results

### 2.1 Liveweight and Instance Segmentation Annotation for Pigs

As public datasets containing both liveweight measurements and corresponding pig images are currently lacking, a standardized method for evaluating various noncontact pig liveweight measurement techniques has not been established. To address this gap, the LISAP dataset was developed and is now publicly available for research purposes. The LISAP dataset includes weight data obtained through manual weighing and corresponding 2D images with annotated instance segmentation masks. The pigs were recorded throughout their fattening periods without any constraints.

**Figure 2:**
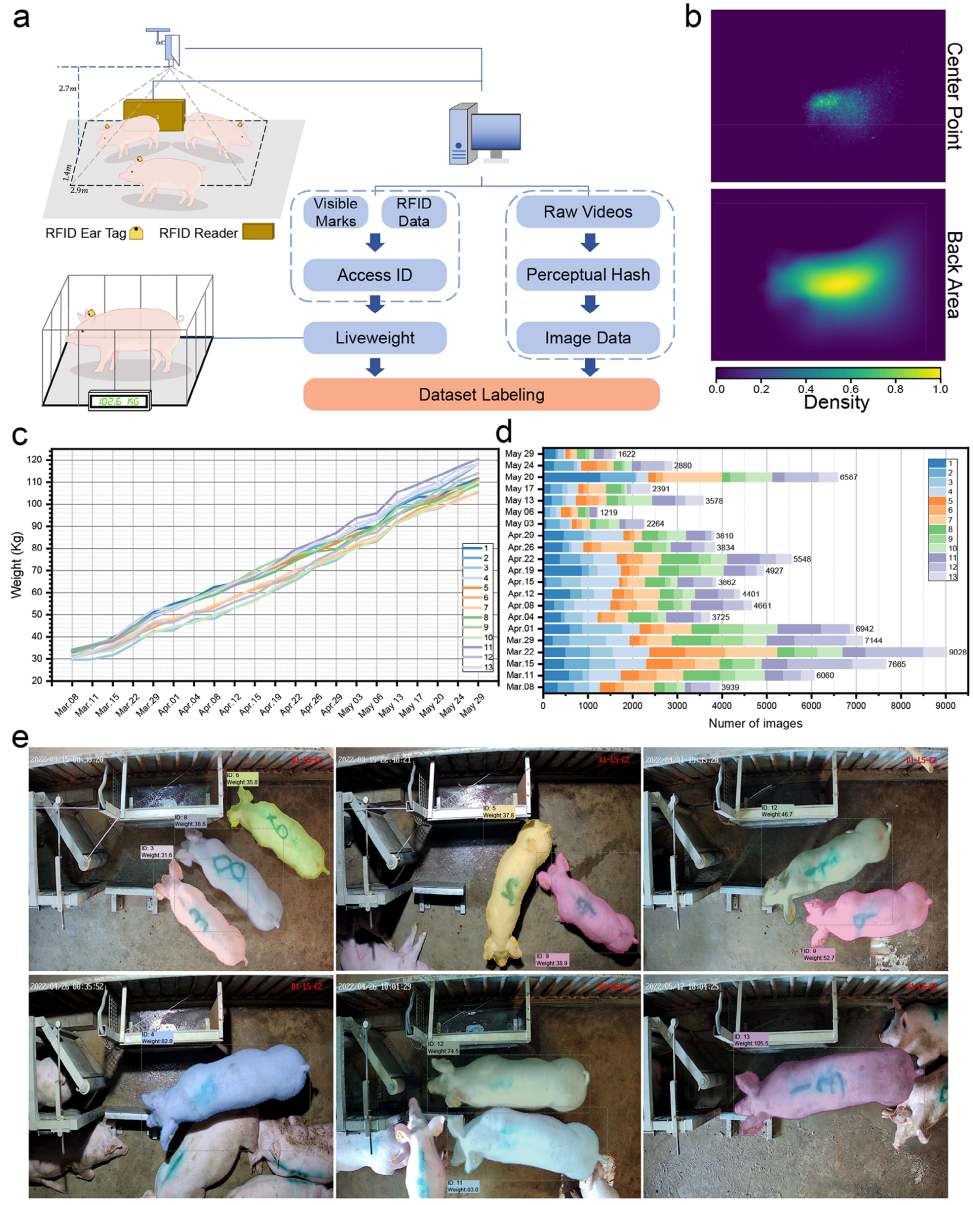
Overview of the LISAP dataset. a) Diagram illustrating the process used to acquire liveweight and image data. Visible marks and RFID information were combined to identify the pigs in the images. b) Density maps showing the distributions of pig centre points and back areas within the images. c) Changes in the liveweights of the pigs over time, with 13 pigs manually weighed once or twice a week, resulting in 273 weight records. d) Distribution of the number of images captured for each pig on different dates, with a total of 96,087 images included in the dataset. e) Examples of annotated images, including their pig IDs, liveweights, and segmentation results.

**Figure** **2**a illustrates the workflow used to acquire the dataset. A radio frequency identification (RFID) reader was placed at the edge of the experimental pig pen to capture the ID information of passing pigs, whereas an overhead 2D camera recorded images. By combining the RFID data with visible markers (Arabic numbers painted on the pigs' backs), the ID of each pig was matched to the corresponding images. Since the camera captured images at approximately 13 frames per second (FPS), resulting in redundant data, similar images were filtered via perceptual hashing (pHash).^[^ ^17^ ^]^ Manual weighing was performed once or twice a week, and the dataset was annotated with the respective pig IDs and their liveweights.

The presence of feeding equipment meant that most pigs appeared mainly in the right and bottom portions of the images. Figure 2b shows the density maps of the pig centre points and back areas within the images. On March 8, 2022, 13 pigs were contained in the experimental pen, with an average weight of 33.1 kg and a standard deviation (SD) of 1.55 kg. By the end of the experiment on May 29, 2022, the average weight of the pigs had increased to 112.5 kg, with an SD of 4.94 kg (see Table S1, Supporting Information).

Throughout the experiment, 21 manual weight acquisition steps were conducted, resulting in 273 weight records, as depicted in Figure 2c. After filtering similar images (refer to Figure S4, Supporting Information), 96,087 images were deemed suitable for inclusion in the dataset. Figure 2d shows the distribution of the images of each pig per day. Variability was observed in the number of usable images collected daily due to hardware issues (e.g., network problems) and pig size differences. The collected images were subsequently annotated, as shown in Figure 2e. Only pigs with clearly visible and complete contours were labeled, with particular emphasis on their backs (see Figure S5, Supporting Information).

The final LISAP dataset consisted of 96,087 images, each of which was annotated with instance segmentation labels and corresponding liveweights. The images were divided into training, validation, and testing sets through stratified sampling on the basis of their dates and pig IDs, with 57,727 images allocated to the training set, 19,228 assigned to the validation set, and 19,332 included in the testing set.

### 2.2 Utilizing Positional and Geometric Information to Perform Liveweight Prediction via the CNN Approach

**Figure 3:**
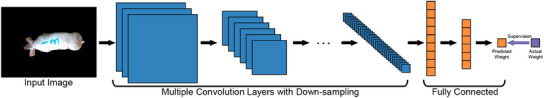
The model architecture that is commonly used for liveweight estimation in CNN‐based approaches. Initially, CNNs iteratively convolve and downsample images to extract salient features. These features are then passed to a fully connected network, either through flattening or by applying global average pooling. The fully connected network estimates the liveweight on the basis of the learned features and their complex relationships within the data.

**Figure 4:**
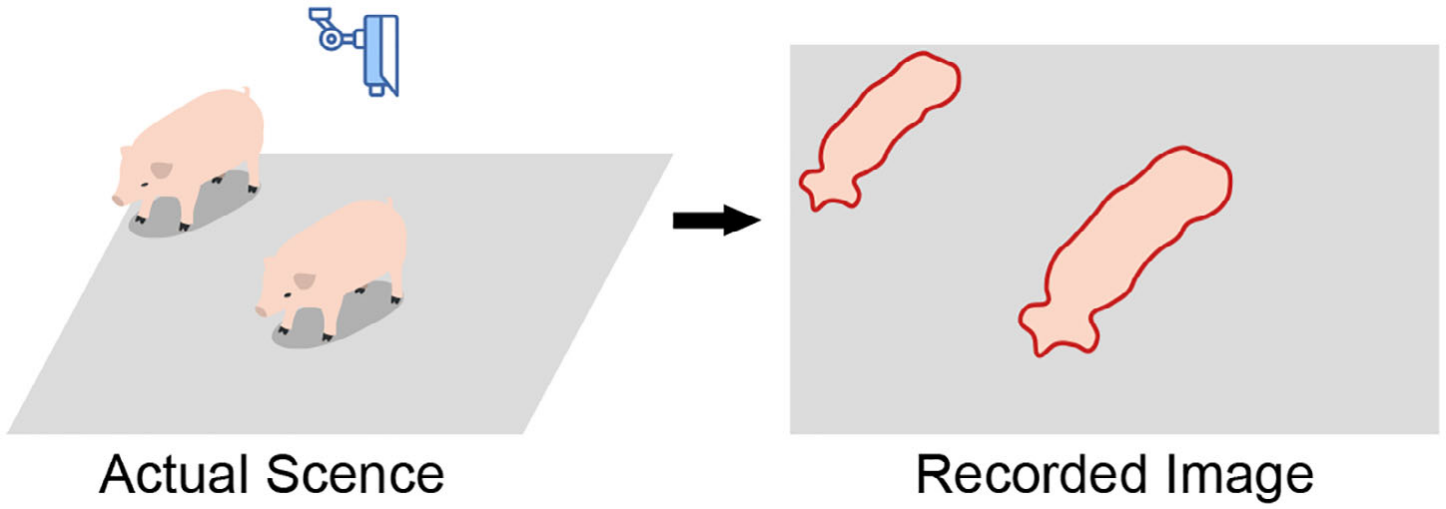
Pig appearance variations based on different positions within the field of view of the camera. Pigs in different locations appear with various sizes in the recorded image, highlighting the influence of their positions on the imaging process.

The CNN approach has been extensively applied to pig weight estimation tasks. **Figure** **3** illustrates the abstract structure of a typical CNN used for this purpose. Since cameras are generally positioned more than 2 m above the target pig pen, the perspective of the camera introduces a notable correlation between the pigs' body shapes and their spatial positions within the pen, as depicted in **Figure** **4**. This correlation highlights the critical role of positional information in predicting pig weights from images. While convolutional operations are inherently translationally invariant and do not directly capture positional changes, research has demonstrated that CNNs can implicitly learn and encode positional information.^[^ ^18^ ^]^ Consequently, CNNs leverage this positional information during liveweight estimation. Furthermore, a detailed morphological analysis of pigs has suggested that the majority of the features that are relevant for weight estimation are derived from geometric structures rather than textures. This underscores the importance of integrating comprehensive geometric information to increase the precision of weight prediction, as textures play a minimal role in this context. Together, both positional and morphological factors are critical for accurately estimating liveweights, supporting the hypothesis that CNNs extract and utilize these key aspects to attain improved performance.

**Figure 5:**
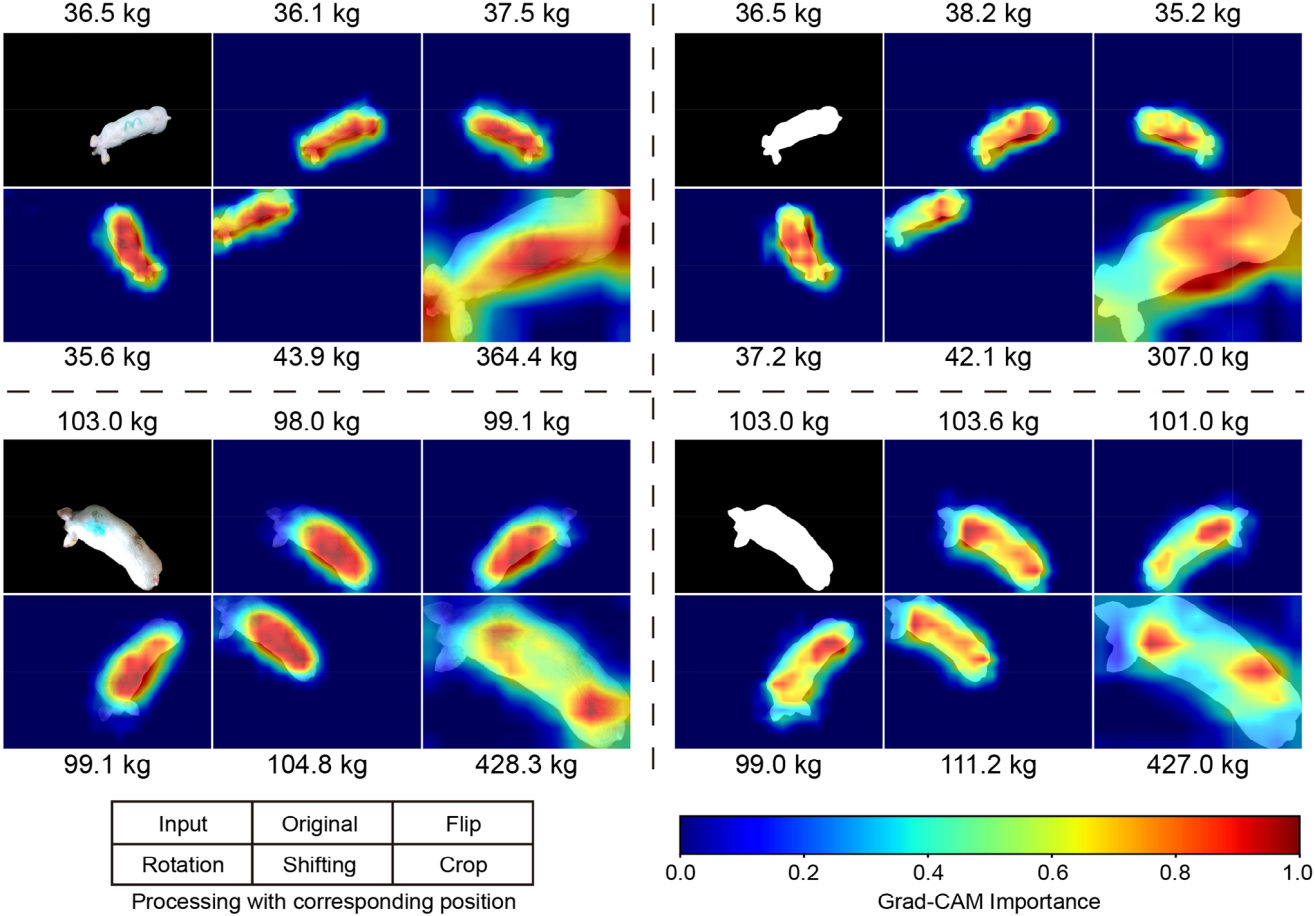
Visualization of the liveweight prediction results obtained via MobileNet with Grad‐CAM. The figure consists of four blocks, each displaying an image processed through different transformations along with the GT or predicted liveweights. The Grad‐CAM heatmaps highlight the areas of each image that the network focuses on when predicting the corresponding liveweight.

To verify our hypothesis, ResNet18^[^ ^19^ ^]^ and MobileNetV2^[^ ^20^ ^]^ were used as the backbone models (i.e., the convolutional components shown in Figure 3) for the neural networks. These models are referred to as ResNet and MobileNet, respectively. Two key experiments were conducted. i) To evaluate the influence of geometric information on network performance, segmented pig images and binary masks were used as inputs during training, validation, and testing. ii) To investigate the role of positional information in the liveweight prediction process, positional alterations were applied during the testing phase, including left‐right flipping, random rotations around the image centres, pig shifting (to the top‐left corner of each image), and cropping each image to fit the corresponding pig's size (see **Figure** **5**).

The quantitative results of the experiments conducted on the test set are shown in **Table** **1**. The networks were trained via ground‐truth (GT) images and evaluated with predictions generated by the CIEN (refer to Section 2.3). In experiment (i), the performance of the network exhibited negligible changes when segmented images, which include comprehensive information, were used instead of binary masks, which contain only geometric information. Notably, MobileNet demonstrated improved predictive performance when using binary masks, with reductions of 0.09 in the mean absolute error (MAE), 0.12 in the mean squared error (MSE), and 0.15% in the mean absolute percentage error (MAPE). This finding indicates that core geometric features provide sufficient information for maintaining comparable predictive performance. Experiment (ii) revealed that applying transformations to the test data, such as flipping and rotation, which preserved the relative positions of elements, did not result in significant changes in the performance of either ResNet or MobileNet. However, when binary masks were used with shifting, ResNet experienced considerable increases in the MAE (12.07), MSE (104.33), and MAPE (14.45%), alongside a notable decrease in the R^2^ score from 0.99 to 0.825. These results highlight the sensitivity of the network to changes in the positional layout of the input data, particularly when such alterations involve displacements or modifications to the original field of view.

**Table 1:** The quantitative liveweight prediction results obtained by the CNN.

Backbone	Processing*	Segmented Image**				Binary Mask**			
		MAE	MSE	MAPE	R^2^	MAE	MSE	MAPE	R^2^
ResNet	Original	1.76	4.91	2.49	0.992	1.79	6.05	2.45	0.990
	Flipping	1.59	4.28	2.24	0.993	1.67	5.43	2.25	0.991
	Rotation	1.56	4.11	2.23	0.994	2.14	7.64	2.93	0.988
	Shifting	8.05	68.32	12.93	0.892	10.28	110.38	16.90	0.825
	Crop	365.08	1.35*e*5	593.02	−211.933	362.49	1.32*e*5	601.14	−208.388
MobileNet	Original	1.84	5.67	2.51	0.991	1.75	5.79	2.36	0.991
	Flipping	1.52	4.13	2.08	0.993	1.61	5.06	2.19	0.992
	Rotation	1.71	4.88	2.34	0.992	1.77	5.90	2.43	0.991
	Shifting	8.68	79.55	14.33	0.874	8.24	72.75	13.72	0.885
	Crop	417.84	1.76*e*5	683.04	−278.101	362.27	1.33*e*5	580.28	−209.425

*The processing are performed during the testing stage. **This indicates the network taking as input during the training, validating, and the testing stage. Networks are trained using GT images, and tested using the results obtained by CIEN.

Our experiments explored two primary dimensions: the impact of geometric information and the influence of positional changes. First, the performance of networks trained with segmented pig images was analyzed in comparison with that of networks trained with binary masks, which contained more focused geometric information. The performance of the CNNs remained consistent across these different input types, with MobileNet showing a slight improvement in performance when binary masks were used. This finding suggests that geometric features—such as contours and body shapes—are adequate for effectively estimating weights. The results align with the notion that texture plays a minimal role relative to that of geometric information, thereby emphasizing the importance of geometric features in CNN‐based liveweight estimation tasks. Second, the role of positional information was investigated by applying various transformations, including flipping, rotation, and shifting, to the test data. Changes in positional information, especially those affecting the field of view and spatial layout, significantly affected the resulting network performance. Specifically, when the positional layout was altered, ResNet exhibited increased errors and a decrease in its R^2^ scores, highlighting its sensitivity to positional information changes. This sensitivity underscores the critical importance of maintaining a consistent positional context to ensure accurate predictions.

To further investigate the CNN's reliance on different features, we tested the MobileNet model trained on segmented images using three distinct test setups, as visualized in Figure S1a (Supporting Information). In the first setup, all pixels were replaced with their mean value, effectively removing texture features while preserving geometric and positional information. The second setup repositioned the pigs to the top‐left corner, altering positional information without affecting geometry or texture. The third setup applied morphological erosion, which shrank the pigs' boundaries and modified geometric information while retaining positional and texture features. The results of the comparison between predicted and actual weights, as presented in Figure S1b (Supporting Information), indicated that removing texture features had minimal impact on prediction accuracy, with the R^2^ value decreasing only slightly from 0.991 to 0.987. This suggests that although the segmented images contain texture information, the network did not rely heavily on this feature for predicting pig weight. In contrast, altering positional and geometric information led to a significant reduction in accuracy, with R^2^ dropping to 0.874 and 0.61, and MAPE increasing to 14.33 and 21.53, respectively. Regression analysis, as illustrated in Figure S1c (Supporting Information), further revealed that the first processing method had minimal impact on the network's predictions. However, the second method caused the network to overestimate pig weight, while the third method led to underestimation. These findings confirm that CNNs primarily rely on positional and geometric information for accurate weight prediction.

Furthermore, gradient‐weighted class activation mapping (Grad‐CAM)^[^ ^21^ ^]^ was employed to visualize how the focus of the network shifted with varying inputs and processing methods, as shown in Figure 5. Manipulations of the images, which altered the positions of objects, significantly influenced the focus of the network, leading to corresponding predictive performance changes. This observation highlights the heightened sensitivity of the network to the manipulation of positional information within the input data, underscoring the importance of preserving the integrity of the positional layout for achieving optimal performance. It was evident that processing variations, which altered the positions of objects within the images, resulted in shifts in the focus of the network. This underscored the sensitivity of the attention paid by the network to spatial information changes within the input data. When binary masks were used as inputs, the attention of the network exhibited a more diffuse and evenly distributed focus, with a particular emphasis on the localization of edges. This suggested that the introduction of the binary mask led the network to prioritize boundary information, causing a shift in its focal point toward the edges. Overall, both the quantitative and qualitative results indicate that the CNN approach relies on both geometric and positional information to predict pig weights.

### 2.3 Contour Information Extraction Network

#### 2.3.1 The Proposed CIEN

**Figure 6:**
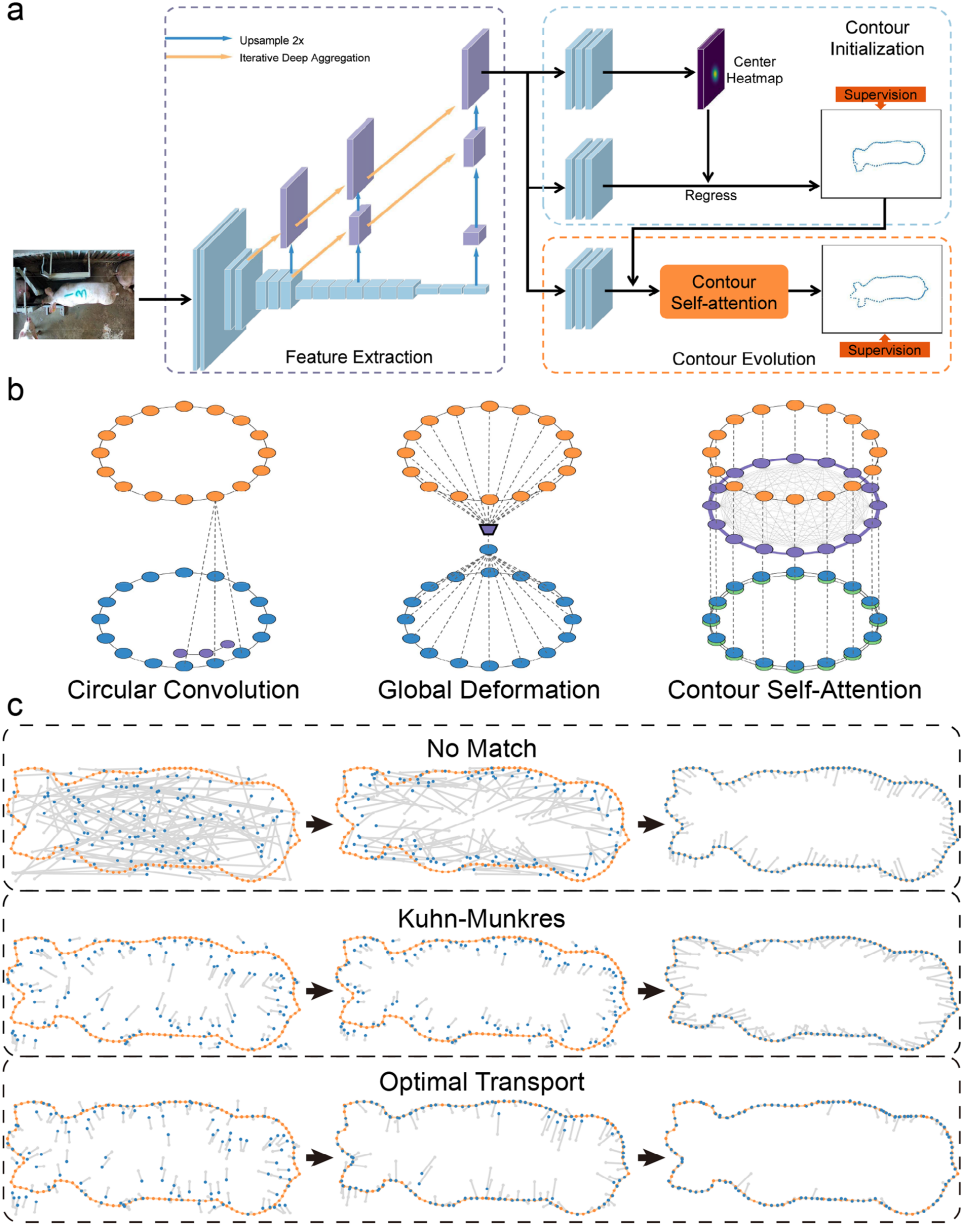
The contour information extraction network used in this study. a) Architecture of the CIEN. b) Comparison among different methods for aggregating point features to predict offsets. The blue points represent point features, the yellow points indicate offsets, the purple dots denote the local kernel function for implementing a circular convolution, the purple trapezoids represent the MLP in the global deformation processes, and the purple and green rings with points signify the MHSA and CPE mechanism, respectively. c) Movement directions of the predicted points during the iterative procedure of the network with different supervision strategies. The yellow points represent GT points, the blue points represent predicted points, and the grey points indicate the previous positions and movement paths.

In this study, we propose a network called the CIEN, which was specifically designed for delineating pig contours in images. Although this task falls under the broader category of instance segmentation, the CIEN was tailored to perform this task without the need for classification. Given the straightforward environment of pig farms and the emphasis on speed and efficiency, the development process focused on a contour‐driven approach that prioritizes both agility and performance. **Figure** **6**a illustrates the architecture of the CIEN, and the pseudocode for the CIEN is shown in Table S2 (Supporting Information). The main steps of the CIEN are described below.

**Feature extraction**: For feature extraction, MobileNetV2 is integrated with the iterative deep aggregation (IDA)^[^ ^22^ ^]^ strategy, enabling the efficient extraction of key features from input images while systematically aggregating them. This enhances the representational power of the model and captures the contextual information that is necessary for precise contour delineation. During the contour initialization process, a learnable architecture inspired by E2EC^[^ ^23^ ^]^ is employed, which directly regresses each contour from the center point to the contour vertex. Although this initialization scheme is coarse, it provides a solid foundation for further refinement.
**Coarse contours**: To refine coarse contours, a contour self‐attention (CSA) mechanism is proposed, as illustrated on the right side of Figure 6b. While DeepSnake^[^ ^24^ ^]^ employs circular convolution to aggregate the features derived from neighboring points and capture global information (see the left part of Figure 6b), this approach requires multiple convolutional layers and downsampling operations, which can limit its effectiveness in terms of learning long‐range dependencies. Conversely, E2EC employs global deformation by flattening and concatenating the features acquired from all points and predicting offsets via a multilayer perceptron (MLP; see the middle part of Figure 6b). However, it neglects the positional relationships between points, leading to inadequate spatial information utilization.
  Our CSA mechanism addresses these challenges by capturing the intricate dependencies among individual points and integrating global features. A central component is contour positional embedding (CPE), which provides each point with positional awareness for distinct identification within the contour. Multihead self‐attention (MHSA)^[^ ^25^ ^]^ is then used to dynamically learn and establish global contextual relationships among these positioned points. This approach, which is enhanced by global information fusion, enables precise predictions to be obtained for the positional offsets of each point, leading to a more refined contour evolution trend.
**Supervision**: Previous studies have typically framed the contour supervision task as a matching problem between two sets of points.^[^ ^23^, ^24^, ^26^ ^]^ This approach, which is referred to as the “HardAssign” strategy, enforces a one‐to‐one correspondence between each predicted point and the corresponding GT point. Consequently, the network training process focuses on iteratively minimizing the distances between these matched points. However, the HardAssign strategy often imposes restrictive constraints on the learning process. It compels the network to concentrate exclusively on these preselected points, neglecting the broader context and intricate characteristics of the entire contour. Since the GT points sampled from a contour represent only a fraction of its complete geometry, strict adherence to these points can lead the network to erroneously focus solely on these specific annotated locations, thereby impeding its ability to effectively conduct learning. For comparative purposes, the Kuhn‐Munkres (KM) algorithm^[^ ^27^ ^]^ is employed to implement the HardAssign strategy.
**Optimization**: In this study, the supervisory task in contour prediction is reframed as a transportation problem that optimizes the alignment between the distributions of the predicted and GT contours. This novel approach, which is referred to as “SoftAssign,” acknowledges that both the predicted and GT points are samples drawn from their respective distributions. The neural network training process is viewed as an iterative refinement of the predicted distribution to align it with the GT distribution. Optimal transport (OT) theory^[^ ^28^ ^]^ is used to minimize the cost of transporting one distribution to another. This approach enables the network to learn not only the locations of individual points but also the overall shape of the contour. The deformation paths formed from the predicted points obtained by the networks during training under various supervision strategies are illustrated in Figure 6c.

#### 2.3.2 Comparative Results of CIEN

To assess the ability of the CIEN to accurately extract pig contours from images, we evaluated the quality of the predicted masks. In instance segmentation tasks, masks are crucial for defining object boundaries, such as the contours of pigs. The predictions of the CIEN result in unordered sets of contour points because of the utilized OT supervision scheme. Traditional geometric methods, such as concave hulls and triangulation, have been employed in previous research to convert these unordered points into masks.^[^ ^29^ ^]^ However, these methods either provide suboptimal performance or are computationally complex. To address these challenges, we reformulated the problem as a traveling salesman problem (TSP).^[^ ^30^ ^]^ The TSP aims to find the shortest path that visits each point and returns to the starting point, providing an optimal connection order for the points of a contour. We applied an enhanced greedy algorithm to solve this TSP; this algorithm dynamically selects the best path for connecting the points. Given the sensitivity of the algorithm to the choice of the initial point, we mitigated this issue by initiating the search process from every possible point and selecting the path that resulted in the most accurate contour. The optimized sequence of points was then used to create a mask that precisely represented the contour of the corresponding pig. This mask enabled a thorough evaluation of the contour extraction capabilities of the CIEN, ensuring that the quality of the extracted contours was accurately assessed.

**Table** **2** presents a comparison among the segmentation results produced by the proposed CIEN method and other widely used methods, which are classified into mask‐based and contour‐based approaches. The considered performance metrics include the average precision (AP), number of parameters (Params), and FPS. In the mask‐based category, both the Mask region‐based CNN (R‐CNN)^[^ ^31^ ^]^ and YOLACT^[^ ^32^ ^]^ achieved notable AP scores, with Mask R‐CNN reaching 97.15 and YOLACT reaching 98.87. Conversely, among the contour‐based approaches, methods such as DeepSnake,^[^ ^24^ ^]^ DANCE,^[^ ^26^ ^]^ and E2EC^[^ ^23^ ^]^ demonstrated strong performance, with AP scores ranging from 98.75 to 98.88. The proposed CIEN outperformed all the compared methods, with an exceptional AP score of 99.56. Notably, varying the backbone from ResNet101 to ResNet50 in the Mask R‐CNN and YOLACT or from ResNet50 to MobileNet in DANCE resulted in a maximal in AP score reduction of only 0.12. This finding indicates that a large backbone is not necessary for effectively extracting image features in pig production environments. Thus, the CIEN, which uses MobileNet as its backbone and IDA for feature aggregation purposes, achieved high accuracy while maintaining low computational complexity. Overall, the results underscore the superior performance of the CIEN in terms of accurately extracting contour information. With 3.8 million parameters and an FPS of 53.7, the CIEN offers both efficiency and performance benefits, making it suitable for deployment on pig farms.

**Table 2:** Comparison among the instance segmentation results produced by different methods.

Method	Backbone	AP↑	AP^50^↑	AP^75^↑	Params↓	FPS↑
*mask‐based*						
Mask R‐CNN	ResNet50	96.79	98.81	98.81	44.4M	6.9
	ResNet101	97.15	97.85	97.85	63.3M	4.1
YOLACT	ResNet50	98.75	98.99	98.98	30.6M	23.8
	Resnet101	98.87	99.00	98.99	49.6M	20.2
*contour‐based*						
DeepSnake	DLA‐34	98.75	99.01	99.00	26.9M	14.8
DANCE	MobileNet	98.79	98.98	98.98	22.1M	17.3
	ResNet50	98.84	99.00	99.00	44.6M	14.5
E2EC	DLA‐34	98.88	99.01	99.01	29.8M	32.6
CIEN (ours)	MobileNet	**99.56**	**99.92**	**99.92**	**3.8M**	**53.7**

**Figure 7:**
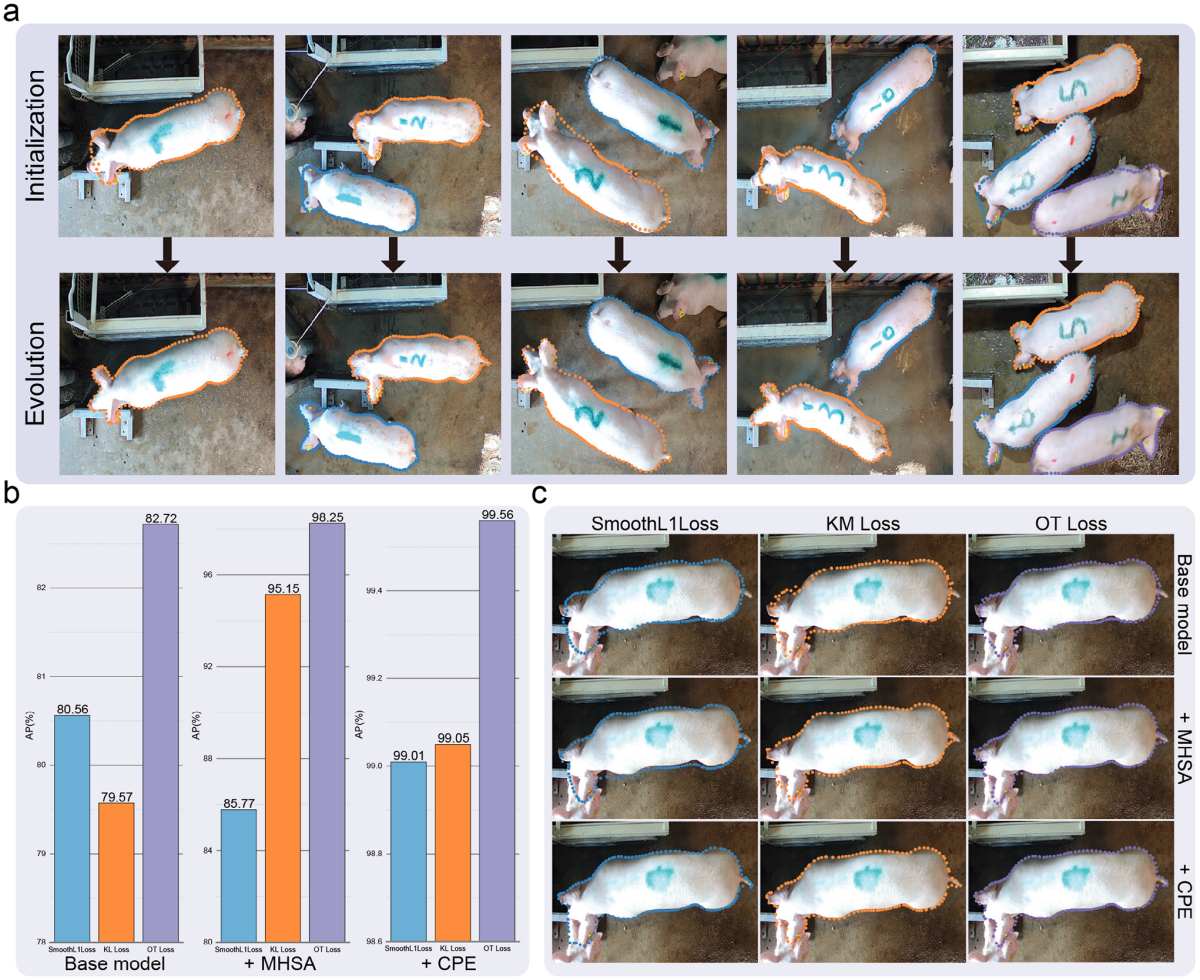
Ablation results obtained for analysing the effects of the different components contained in the CIEN. a) Visualization of the predicted initial contours and the corresponding evolved contours. b) Quantitative results of the improvements yielded by the CSA mechanism and OT loss. c) Visualization of the predicted contours produced with and without the CSA mechanism under different loss functions.

**Figure** **7**a displays examples of the initial and evolved contours. The visualization illustrates that the initial contours were quite coarse in terms of defining the boundary of the pig, emphasizing the necessity of performing contour evolution to acquire a refined result. To quantitatively assess the impacts of the CSA mechanism and the OT loss on the contour evolution process within the proposed CIEN method, an ablation study was conducted. The results of this analysis are presented in Figure 7b. The baseline model included only the feature extraction and contour initialization components. The data show that incorporating both the MHSA and CPE mechanisms during evolution allowed the CIEN to achieve optimal performance. Additionally, the CIEN consistently attained the highest AP scores when supervised by the OT loss. To further assess the improvements offered by the CSA mechanism, we conducted a series of experiments. Without altering the overall structure of the CIEN, we replaced the CSA module with two alternative methods: global deformation and circular convolution. The results, as detailed in Table S3 (Supporting Information), have demonstrated that the CSA mechanism outperformed both alternatives, achieving the highest AP value when OT Loss was applied. Notably, CSA achieved this superior performance while maintaining a low parameter count and high computational efficiency. This experiment also confirmed that the OT Loss consistently enhances model performance across different methods. The visualization results in Figure 7c further demonstrate that the CSA mechanism and OT loss significantly improved the alignment of the predictions with the actual pig outlines, such as the head regions.

### 2.4 Liveweight Estimation Network

#### 2.4.1 The Proposed LWEN

**Figure 8:**
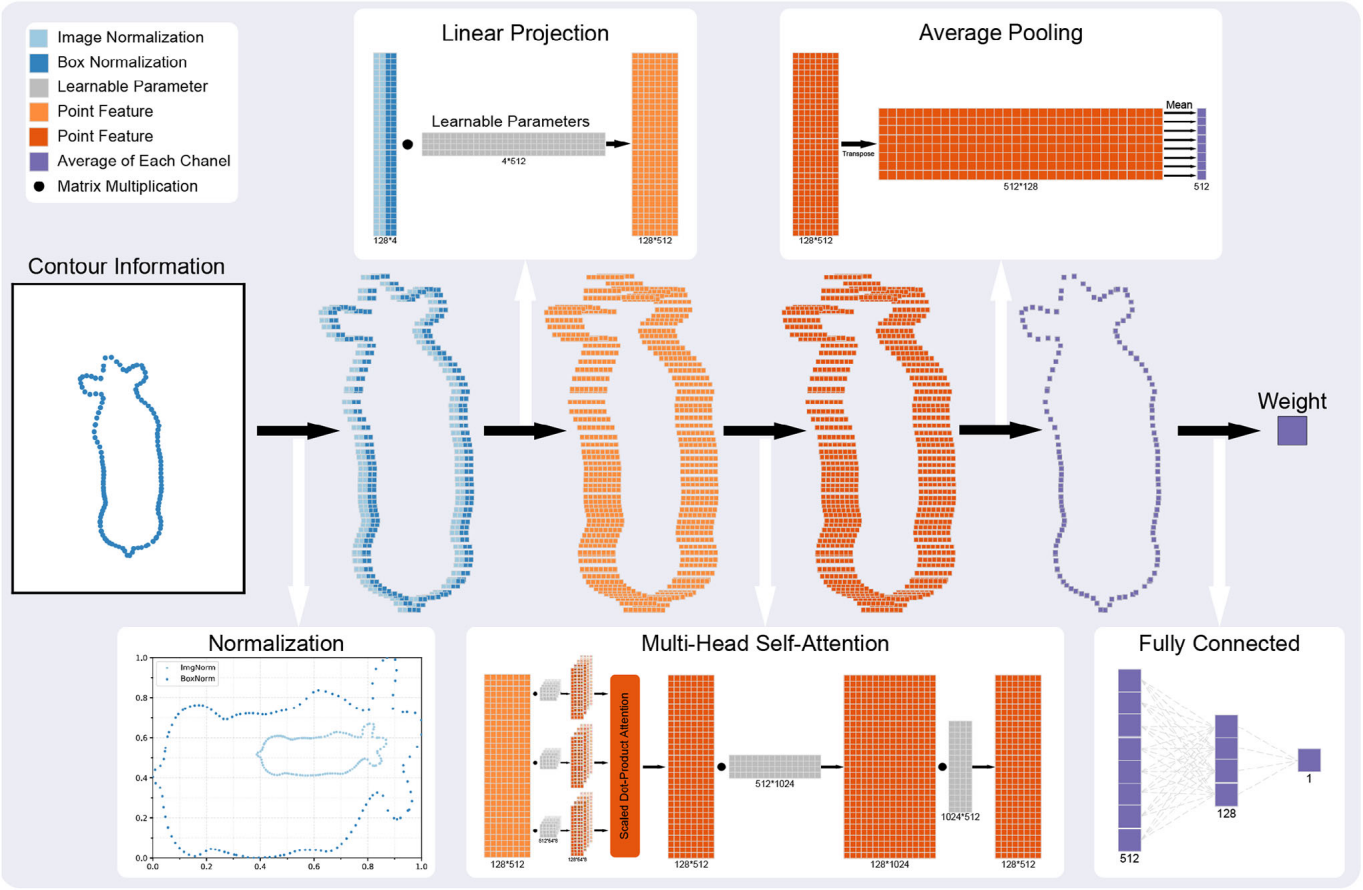
Workflow of the LWEN, which explicitly uses spatial and geometric information for predicting liveweights. The contour information refers to the coordinates of each point that is uniformly sampled from the contour.

Building on the refined contour data provided by the CIEN, we propose a network named the LWEN, which was designed for accurate liveweight prediction. The LWEN method aims to significantly reduce the number of required parameters and increase the inference speed of the model without compromising accuracy. **Figure** **8** illustrates the architecture of the LWEN, which consists of three key components that enable efficient liveweight estimation, and the corresponding pseudocode for the LWEN is shown in Table S4 (Supporting Information).

The LWEN comprises three key components that facilitate efficient liveweight prediction. Initially, the coordinates undergo normalization relative to both the input image and the bounding box (image and box normalization), ensuring consistency across the data. These normalized data are subsequently transferred to a higher‐dimensional space through linear projection, enabling the extraction of richer features. Crucially, the relationships among these projected features are learned and aggregated, and this task was accomplished in this study through the utilization of an MHSA layer. Finally, liveweight estimation is achieved by averaging the features across channels and regressing the output through a fully connected layer. The detailed workflow of the LWEN is described in Section 4.

#### 2.4.2 Analysis of the Proposed LWEN

To assess the performance of the LWEN, the model was trained on the LISAP dataset and evaluated on contours generated by the CIEN. To benchmark our approach against CNN‐based methods, we selected ResNet and MobileNet for comparison, utilizing segmented images, binary masks, and contour information in this study. As shown in **Table** **3**, the LWEN demonstrated a compelling combination of low cost, high precision, and exceptional speed, making it an advantageous solution for performing liveweight estimation in dynamic environments such as pig farming, where both accuracy and real‐time performance are crucial.

**Cost Efficiency**: The LWEN struck a notable balance between model complexity and performance, with a parameter count of 3.2 million. This was substantially lower than that of ResNet, which required 11.2 million parameters, and only slightly higher than the 1.9 million parameters required by MobileNet. The reduced parameter count of the LWEN resulted in lower computational and storage costs, rendering it a cost‐effective choice for liveweight estimation.
**High Precision**: The LWEN excelled at delivering precise liveweight estimates, achieving an MAE of 1.45, an MSE of 4.45, and a MAPE of 1.97. The R^2^ value of 0.993 further emphasizes its high accuracy. In comparison, ResNet_1D and MobileNet_1D, which utilize 1D convolutions for processing contour coordinates, exhibited significantly higher MAE and MSE values, indicating lower precision. Despite processing contour coordinates, the MLP demonstrated considerably poorer performance, with an MAE of 42.14 and an MSE of 2436.75.
**Exceptional Speed**: The speed of the LWEN is a standout feature, enabling it to reach 1131.6 FPS, which was significantly faster than other methods were. Although the MLP yielded an impressive FPS of 3388.1, it failed to provide accurate liveweight estimates, underscoring the trade‐off between speed and precision. This finding demonstrates that the LWEN effectively balances both speed and accuracy, making it a superior choice for liveweight estimation.

**Table 3:** Quantitative results for liveweight estimation produced by different methods.

Method	Input	MAE	MSE	MAPE	R^2^	Params	FPS
ResNet	Segmented Image	1.76	4.91	2.49	0.992	11.2M	286.7
MobileNet	Binary Mask	1.75	5.79	2.36	0.991	1.9M	186.5
ResNet_1D	Contour Coordinates	10.31	170.06	14.25	0.731	3.9M	242.8
MobileNet_1D	Contour Coordinates	14.54	337.42	18.54	0.466	1.8M	149.0
MLP	Contour Coordinates	42.14	2436.75	53.20	−2.854	5.3M	3388.1
**LWEN**	Contour Coordinates	1.45	4.45	1.97	0.993	3.2M	1131.6

**Note**: The “1D” designation refers to methods using 1D convolutions, whereas the other methods utilize 2D convolutions.

The results in Table 3 suggest that directly using contour information for weight prediction with CNN‐based methods is not ideal. While ResNet and MobileNet achieve high accuracy when using segmented images or binary masks, their performance drops significantly when applied to contour data, with R^2^ values decreasing from 0.992 and 0.991 to 0.731 and 0.466, respectively. This is because contour data contains limited local information, whereas many weight‐related features are embedded in long‐range dependencies. For instance, body length strongly correlates with weight, yet head and tail points are spatially distant in the contour representation. Capturing these relationships requires modeling long‐range dependencies, which CNNs struggle with as they primarily focus on local features.

MLP performs poorly in this task likely because, although it has the potential to capture global features, it lacks the ability to explicitly model long‐range dependencies. This is evident in its contrasting performance: when using ground truth contour data, MLP achieves an MAE of 1.59, MAPE of 2.14, and R^2^ of 0.991. However, when relying on CIEN‐predicted contour data, its R^2^ drops drastically to ‐0.285. This suggests that MLP primarily learns simple arithmetic operations involving specific values rather than identifying complex structural patterns in the data. In contrast, LWEN's design effectively captures global information and models long‐range dependencies through the MHSA mechanism, making it highly effective for liveweight estimation using contour information.

We attribute LWEN's high prediction accuracy and exceptional speed to its direct utilization of contour information for liveweight estimation. After normalization, the network requires only six matrix multiplications to produce the final weight prediction. To explore its scalability, we extended LWEN by increasing its width and depth, resulting in two variants: LWEN‐Wide and LWEN‐Deep:

- LWEN‐Wide expands the number of feature channels after linear mapping from 256 to 1024.
- LWEN‐Deep increases the number of MHSA layers from 1 to 6.

As shown in Table S5 (Supporting Information), these modifications slightly improved prediction accuracy, with MAE decreasing to 1.39 (LWEN‐Wide) and 1.36 (LWEN‐Deep), and R^2^ improving to 0.994. However, these enhancements came at the cost of reduced processing speed. The FPS of LWEN‐Wide and LWEN‐Deep dropped to 1088.6 and 207.6, respectively, compared to 1131.6 for the original LWEN. These results suggest that LWEN's efficiency is largely due to its minimal matrix multiplications. Furthermore, they demonstrate LWEN's strong scalability–adjusting its width and depth can enhance prediction accuracy when needed, albeit with trade‐offs in speed.

**Figure 9:**
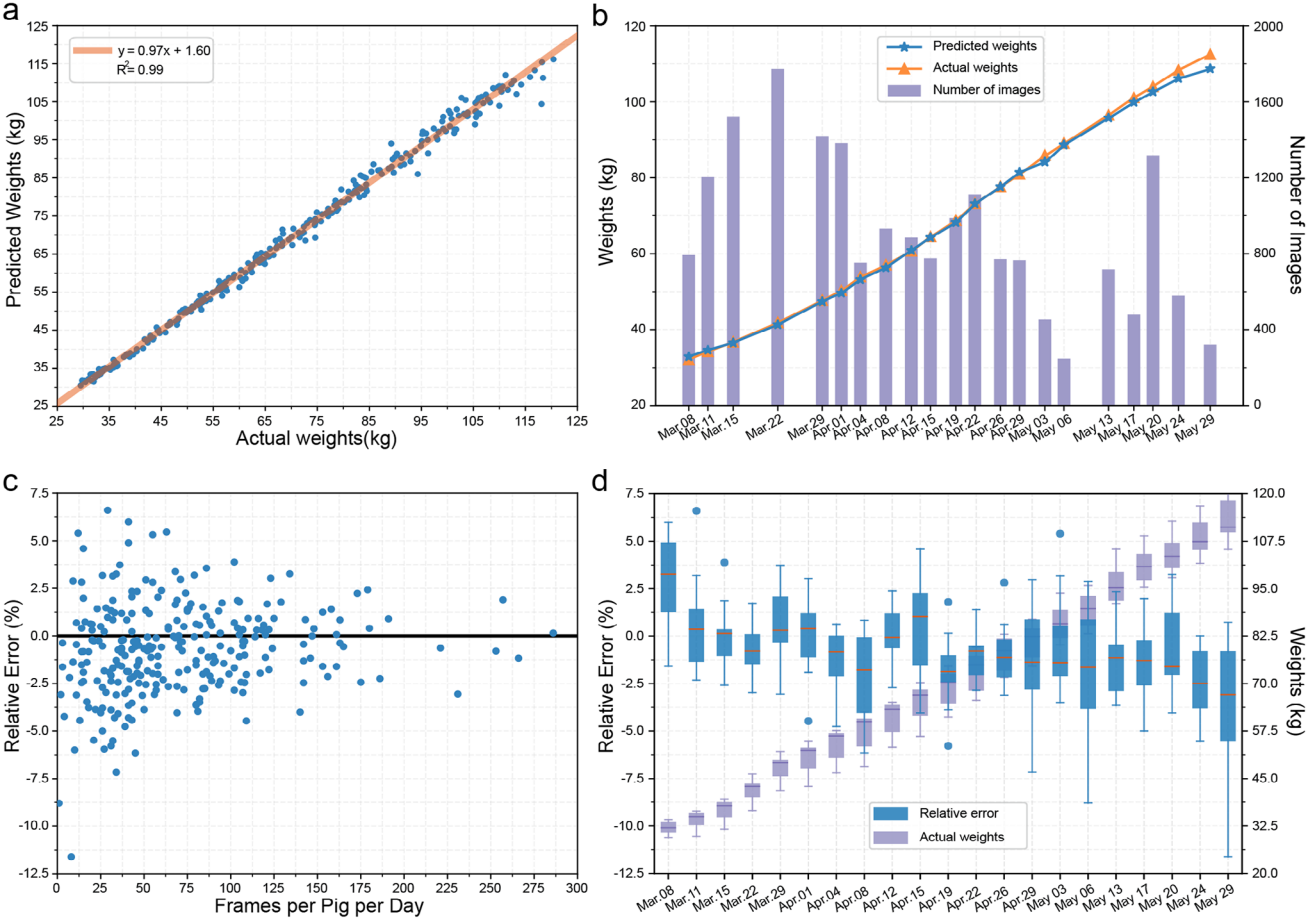
Statistical results yielded by the LWEN. a) Relationships between the actual and predicted weights. b) Average actual and predicted weights versus the number of test images captured per day. c) The relative prediction error as a function of the number of test images acquired per pig per day. d) Distribution of the relative error with increasing pig growth.

**Figure** **9**a demonstrates the strong correlation between actual and predicted weights, highlighting the accuracy of LWEN. Figure 9b presents the pen‐level predictions alongside the number of test images captured per day, showing that LWEN maintains high accuracy throughout the pigs' growth period and is largely unaffected by the number of images. Figure 9c shows that the relative error decreased as the number of frames per day increased, indicating that more frequently acquiring images helps attain improve accuracy. In Figure 9d, we observe a consistent trend of slight underestimation as the pig weights increased, highlighting the ability of the model to generalize well across different weight distributions.

Specifically, we conducted a Shapiro‐Wilk test on the relative error distributions for each day, and the results indicated that these distributions follow a normal distribution. Additionally, a Levene test confirmed that the relative error distributions across the days exhibited homogeneity of variance, further supporting the robustness and reliability of our model. However, we observed that as pigs grow heavier, the prediction error tends to increase slightly, particularly for those with higher weights. After performing a least significant difference (LSD) post‐hoc test following an analysis of variance (ANOVA), we found that the mean relative prediction errors on May 29 (with actual liveweights ranging from 105.3 kg to 120.4 kg) differed significantly from those on other days, except for April 15. This suggests that the framework performs somewhat less accurately in higher weight ranges.

To assess the generalizability and robustness of our framework, we conducted additional experiments in two supplementary pens (SP‐1 and SP‐2), located on the same farm as the experimental pen (EP) in the LISAP dataset. SP‐1 and SP‐2 contained 13 and 12 pigs, respectively, with their image counts and weight distributions shown in Figure S2a of the Supporting Information. The framework was trained exclusively on the EP training set, without incorporating any data from SP‐1 or SP‐2, which allowed us to evaluate the cross‐pen generalization of the framework. As illustrated in Figure S2b (Supporting Information), our framework maintained strong performance in both SP‐1 and SP‐2, achieving R^2^ values exceeding 0.98 in both cases. In comparison to CNN‐based methods (MobileNet and ResNet), our framework consistently demonstrated lower MAE and MAPE while maintaining a high R^2^ score. Furthermore, regression analysis between actual and predicted weights, as illustrated in Figure S2c (Supporting Information), confirmed the strong generalization capability of our model, as data points in both test scenarios clustered tightly along the ideal fit line. These findings validate the high predictive accuracy of our approach across different pens and highlight its superiority over CNN‐based architectures in pig weight estimation.

While LWEN performs satisfactorily in both SP‐1 and SP‐2 configurations, its prediction accuracy slightly decreases compared to the EP. This discrepancy is primarily attributed to variations in camera configurations, including differences in installation height (ranging from 271.9 to 273.9 cm) and angular orientation (varying by approximately 5 degrees) across the three cameras, as well as the lack of calibration. As shown in Figure S3 (Supporting Information), these installation parameters significantly impact swine imaging outcomes. Cameras mounted at lower heights capture larger relative swine dimensions, whereas higher‐mounted cameras produce the opposite effect, leading to variations in estimated pig morphology. Additionally, slight differences in tilt angles – resulting from the inability to ensure perfect perpendicularity during installation – may alter the proportion of the side body surface captured, further affecting imaging accuracy. Inherent differences in camera specifications, such as focal length and sensor quality, also introduce varying degrees of imaging distortion, influencing weight estimation performance. Together, these factors lead to subtle differences in captured pig morphology, ultimately reducing weight estimation accuracy. These findings emphasize the need for standardized camera deployment in pig weight estimation systems. Consistent installation heights, angular orientations, and thorough calibration procedures are crucial for ensuring reliable measurement consistency.

In comparison to previous methods, our proposed approach demonstrates significant improvements in both efficiency and accuracy. For example, He et al.^[^ ^15^ ^]^ developed a regression neural network to estimate pigs' liveweights from 2D images, achieving a speed of 143 FPS. However, their method is limited by its accuracy, with an MAE of 6.37, which reduces its applicability for precise livestock management. Meanwhile, Kwon et al.^[^ ^10^ ^]^ extracted multiple body parameters from point clouds of pigs and input them into an MLP, achieving an R^2^ value of 0.953. However, this method suffers from limited speed, operating at only 2.5 FPS, making it unsuitable for real‐time applications. In contrast, LWEN offers a significant improvement in speed without sacrificing accuracy. Our framework achieves a speed of 1131.6 FPS, with an R^2^ value of 0.993 for predicted weight. Furthermore, LWEN is more parameter‐efficient, with only 3.2 million parameters, compared to the 55.0 million parameters in the method proposed by Ref. [13], which achieved an MAE of 3.237 and an R^2^ value of 0.742 for its predicted results using a modified vision transformer.

**Figure 10:**
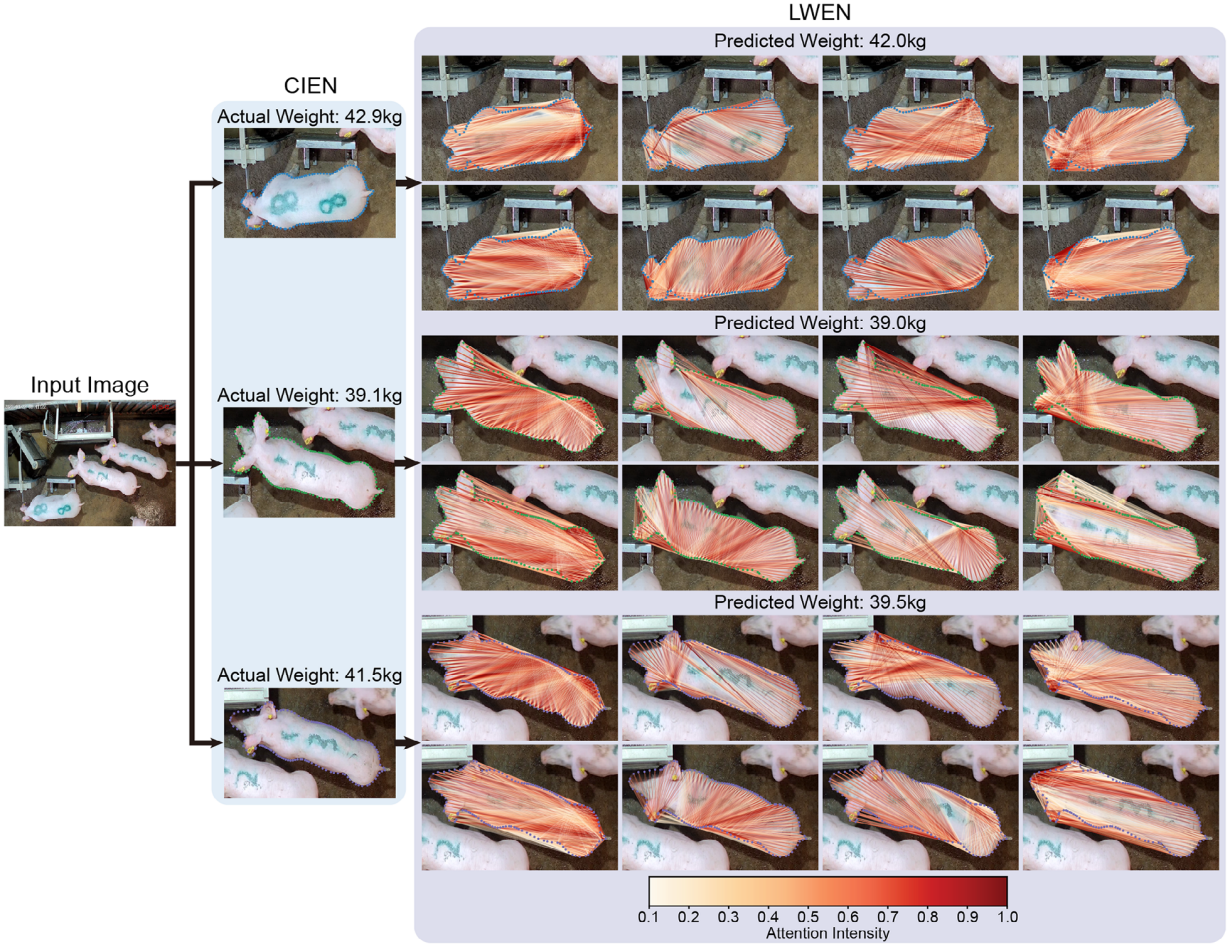
Visualization of the characteristic patterns captured by each head in the MHSA layer of the proposed network. The attention intensity was normalized to a range of 0.0 to 1.0 at each point, with intensities below 0.1 excluded to provide improved visualization clarity.

To provide a more intuitive understanding of the effectiveness of the LWEN, **Figure** **10** visualizes the obtained results. After the CIEN generated contour information, the LWEN processed and aggregated the data through MHSA. Each attention head within the MHSA mechanism captures specific relationships between different body parts. For example, the fifth head (the first item in the second row for each pig) specializes in feature patterns related to body length, whereas the sixth head focuses on body width. This visualization demonstrates how each head contributes uniquely to the overall liveweight estimation process.

## 3 Discussion

In this study, we demonstrated the mechanisms that enable neural networks to predict liveweights and proposed a novel dual‐network framework for accurately and efficiently estimating the liveweights of pigs from 2D images. Our framework was validated to show that it enables real‐time liveweight monitoring and prediction for unconstrained animals while requiring minimal hardware resources.

We explored the impact of positional and geometric information on the CNN‐based liveweight prediction process. Our findings highlight the significance of these factors in attaining improved prediction accuracy and addressing the challenges associated with estimating pig weights from 2D images. The results confirm that CNNs benefit from incorporating both positional and geometric data. While CNNs can adapt to varying levels of positional information, maintaining a consistent and accurate positional context is essential for achieving optimal performance. These insights deepen the understanding of how CNNs utilize spatial and geometric features, offering valuable guidance for advancing noncontact liveweight measurement technologies.

To effectively leverage these insights, we designed two distinct networks as part of a unified framework, i.e., the CIEN and LWEN, each of which address specific challenges encountered during the liveweight estimation process. Each network was tailored to optimize specific aspects of the overall framework, ensuring high accuracy, efficiency, and real‐time performance.

The CIEN was developed to address the challenge of accurately extracting contours from images. An effective contour extraction method is crucial for liveweight estimation, as it provides the foundational data needed for subsequent analysis tasks. Traditional contour extraction methods often struggle with coarse and imprecise boundaries, significantly impacting the accuracy of the resulting liveweight predictions. The CIEN addresses this issue by integrating a CSA mechanism and an OT loss. CSA allows the model to focus on the most relevant parts of a contour, enhancing its extraction precision. The OT loss ensures that the predicted contours closely align with the actual object boundaries, leading to a more accurate representation of the shape of the target pig. By employing the MHSA and CPE mechanisms, the CIEN captures complex relationships between contour points, producing highly refined contours that are essential for accurately estimating liveweights.

To transform the refined contours produced by the CIEN into precise liveweight estimates, the LWEN was specifically designed for this task. By focusing on processing these contours with high accuracy and minimal computational costs, the network was optimized exclusively for liveweight prediction, making it independent of the contour extraction process. With its compact architecture consisting of 3.2 million parameters, the LWEN algorithm strikes a balance between model complexity and performance, ensuring cost‐effectiveness while maintaining high precision. Its impressive speed of 1131.6 FPS makes it highly suitable for real‐time applications in dynamic environments. Furthermore, the network achieved an R^2^ value of 0.993, enabling it to efficiently process and integrate contour information, thereby delivering accurate liveweight predictions.

The separation of the proposed framework into two networks ‐ the CIEN for contour extraction and the LWEN for liveweight estimation allows each network to specialize in its respective task. The CIEN focuses on achieving improved contour accuracy, which is critical for accurately predicting liveweights, whereas the LWEN builds on this accuracy to provide precise liveweight estimates in a computationally efficient manner. This modular approach not only enhances the overall performance of the framework but also allows for the targeted optimization of each component, leading to better results than those produced by a monolithic approach.

Together, the CIEN and LWEN represent significant advancements in the liveweight estimation domain. The CIEN refines the contours needed for obtaining accurate predictions, whereas the LWEN ensures that these contours are used efficiently to provide precise liveweight estimates. Crucially, CIEN's output can be directly fed into LWEN without additional computational overhead, allowing seamless integration and ensuring that the system's end‐to‐end latency remains within real‐time constraints. As a result, the proposed framework attains an overall processing speed of 51.7 FPS, which is driven by the LWEN's remarkable computational throughput of 1131.6 FPS, complemented by CIEN's 53.7 FPS processing capability. The combined strengths of these networks offer a powerful solution for real‐time, accurate liveweight measurement in pig farming scenarios, effectively for addressing the challenges related to both accuracy and speed.

In advancing the noncontact liveweight measurement field, the LISAP dataset addresses a critical gap by providing a comprehensive collection of liveweight data paired with corresponding annotated image resources that have been notably absent in public domains. This dataset significantly advances the field by offering a well‐annotated, diverse, and extensive resource, which is crucial for developing and validating new methods. The availability of such a dataset supports the effective training and validation of innovative models such as the CIEN and LWEN, thereby enhancing their precision and applicability. By filling this critical gap, the LISAP dataset not only contributes to the improvement of liveweight estimation techniques but also ensures that these methods can be effectively applied in real‐world scenarios.

With the advancement of modern agriculture, the demand for large‐scale phenotypic data collection is growing, driving the need for non‐contact measurement methods that offer accuracy, speed, and cost‐effectiveness. Although previous studies using depth or 3D imaging for liveweight estimation have shown promising results,^[^ ^10^, ^13^, ^14^, ^33^, ^34^, ^35^ ^]^ they face several limitations. These methods often require complex procedures to extract usable data from raw inputs obtained by depth cameras, leading to long processing times and high computational costs. Additionally, depth cameras are more expensive than standard 2D cameras and have a narrower field of view, which increases hardware costs, especially in large‐scale applications. In contrast, our method, which relies on 2D images, offers a more cost‐effective and efficient solution. It addresses the challenges of 3D imaging while still maintaining high accuracy for liveweight estimation.

However, our method does have some limitations. Specifically, our approach relies solely on top‐view images of the pigs' backs, which limits the framework's ability to capture the full 3D morphology of the animals. Consequently, the framework cannot incorporate other potentially relevant information, such as height and girth, which could influence weight estimation accuracy. Additionally, the 2D images are projections of the pigs' 3D contours onto the imaging plane, meaning that spatial information is not directly captured. This may lead to biased contour information, especially when sampling the same back contour in both the 2D images and the real 3D space. While deep learning technology can adapt to some of these discrepancies, it is likely that these factors could still affect the learning process and prediction accuracy. To mitigate these limitations, future work will focus on incorporating 3D imaging techniques to capture more comprehensive data, extending the CIEN‐LWEN framework to leverage 3D contour information for more accurate phenotypic predictions.

While the proposed framework can predict the liveweight of multiple pigs simultaneously in an image without requiring constraints, its deployment in real‐world environments does face several challenges. Specifically, the study did not account for variations in lighting conditions, different pig breeds, or occlusions. The LISAP dataset was collected from a single breed of pigs in a controlled farming environment and does not include images where pigs are heavily obscured. Consequently, these factors may impact the robustness of the framework in more varied real‐world conditions. In future work, we plan to address these challenges by enhancing the CIEN component to detect occlusions and by expanding the dataset to include images of different pig breeds and diverse environmental conditions.

## 4 Experimental Section

### Production of the LISAP Dataset

The data utilized in this study were collected at the experimental farm of the Center of Quality Test and Supervision for Breeding Swine in Wuhan, China. Thirteen pigs introduced to the experimental pen, with an average starting weight of 33.1 ± 1.55 kg and an average final weight of 112.5 ± 4.94 kg, were the subjects of this study. A standard 2D‐RGB camera with a 6‐mm focal length and a pixel count of 4,000,000 was mounted on the ceiling of the pen at a height of 2.72 m, covering a floor area of 2.90 × 1.40 *m* ^2^.

An RFID reader positioned below the camera and near the side of the pigsty recorded the IDs and timestamps of the pigs as they passed by. The data were then transferred to a MySQL server. Owing to the nature of RFID technology, when multiple pigs were recorded simultaneously, the system was unable to distinguish individual IDs in the same image and could only record their coexistence. As an auxiliary identification method, the pigs were marked on their backs with paint.

Images were captured by the 2D‐RGB camera. To balance storage efficiency with the precision of this study, video streaming was conducted at 13 frames per second. The resulting frames were saved in the PNG format with a resolution of 768 × 576. The pHash algorithm was employed to filter similar adjacent frames (Figure S4, Supporting Information).

i. A given image *I*_*n*_ acquired at frame *n* was converted to a grayscale image $I_{n}^{\prime }$ with dimensions of 32 × 32.
ii. The discrete cosine transform was applied to extract features from $I_{n}^{\prime }$, resulting in a coefficient matrix *C*_*ij*_ (*i* = 0, 1, …31, *j* = 0, 1, …31). The 8 × 8 lower frequency coefficient matrix in the top left $C_{ij}^{\prime }\;\left(i=0,1,\text{…}7,j=0,1,\text{…}7\right)$ was then selected.
iii. The average value μ was calculated from $C_{ij}^{\prime }$. A 64‐bit hash string *H*_*n*_ = {*h*_*n*_(*ij*), *i* = 0, 1, …7, *j* = 0, 1, …7} for *I*_*n*_ was obtained, where
(1) $$h_{n}\left(ij\right)=\left\{\begin{array}{cc} 0 & \text{if}\;C_{ij}^{\prime }\leq \mu \\ 1 & \text{otherwise.} \end{array}\right.$$
iv. The hash string *H*_*n* + 1_ for the adjacent frame *I*_*n* + 1_ was computed in the same manner. The Hamming distance (HD)^[^ ^36^ ^]^ was used to measure the similarity between *H*_*n*_ and *H*_*n* + 1_:
(2) $$\text{HD}\left(H_{n},H_{n+1}\right)=\sum_{i=0}^{7}\sum_{j=0}^{7}h_{n}\left(ij\right)\;\text{XOR}\;h_{n+1}\left(ij\right),$$
(3) ∀HD∈N,0≤HD≤64
v. If HD ⩽ τ, *I*_*n*_ and *I*_*n* + 1_ were considered similar, where τ was empirically set to 15 in this study.

Liveweights were recorded by driving the pigs to the weighbridge at approximately 10 a.m. for each measurement (detailed data are provided in Table S1, Supporting Information). Through this procedure, a total of 417,868 images were initially selected. After similar images were filtered via pHash and unqualified images were removed through visual inspections, 96,087 images were retained for the LISAP dataset. These images were subsequently annotated via EISeg software,^[^ ^37^ ^]^ resulting in instance segmentation masks saved in the COCO format.^[^ ^38^ ^]^ Importantly, only the back area of each pig, which fully appeared in the images, was annotated in this study (e.g., exposed feet and legs were not included, as illustrated in Figure S5, Supporting Information).

### Design and Development of CIEN

*CIEN Architecture* The CIEN was composed of three main components: feature extraction, contour initialization, and contour evolution modules. For feature extraction, the first seventeen layers of MobileNetV2 were utilized as the backbone, and IDA^[^ ^22^ ^]^ was applied to aggregate the information. The IDA function *T* for a series *o*_1_, …, *o*_*n*_ with increasing downsampling scales is formulated as follows:

(4) $$T\left(o_{1},\cdots ,o_{n}\right)=\left\{\begin{array}{cc} o_{1} & \text{if}\;n=1\\ T(N\left(o_{1},o_{2}\right),\cdots ,o_{n}) & \text{otherwise} \end{array}\right.$$

where *N* represents the aggregation node, as depicted in the purple block in Figure 6a. The outputs of MobileNetV2 layers 4, 7, 11, and 17 were used as the inputs of the IDA process, and they are denoted as *o*_1_, *o*_2_, *o*_3_, and *o*_4_ respectively. These selected outputs contained semantic information with different scales: 4‐, 8‐, 16‐, and 32‐fold downsampling. Consequently, a feature map F with dimensions of $\frac{H}{4}\times \frac{W}{4}\times 24$ was obtained through *T*(*o*_1_, *o*_2_, *o*_3_, *o*_4_), where *H* and *W* denote the height and width of each input image, respectively.

For the contour initialization stage, similar to E2EC, two convolution blocks were first applied to F to produce a centre heatmap $F^{ct}\in R^{\frac{H}{4}\times \frac{W}{4}\times 1}$ and a regression map $F^{reg}\in R^{\frac{H}{4}\times \frac{W}{4}\times \left(N\times 2\right)}$, where *N* denotes the number of contour vertices. Let *n* denote the number of objects contained in the given image; the coordinates of the centre points of the objects could be regarded as $\zeta ={\left\{x^{p},y^{p}\right\}}_{p=1}^{n}$. ζ could be obtained via the GT during training or by applying a threshold to $F^{ct}$ during testing. Then, ζ could be used to index $F^{reg}$ along the first two channels ($\frac{H}{4}\times \frac{W}{4}$) to obtain the initial contours ${\left\{{\left\{x_{i}^{p},y_{i}^{p}\right\}}_{i=1}^{N}\right\}}_{p=1}^{n}$.

For the contour evolution stage, a CSA mechanism was proposed to refine the initial contours, as illustrated on the right side of Figure 6b. The process of generating offsets (denoted as {(Δ*x*_*i*_, Δ*y*_*i*_)∣*i* = 1, 2, …, *N*}) for refining a contour point set (denoted as {(*x*_*i*_, *y*_*i*_)∣*i* = 1, 2, …, *N*}) is formulated as:

(5) $${\left\{\left(\Delta x_{i},\Delta y_{i}\right)\right\}}_{i=1}^{N}=\text{CSA}\left({\left\{\left(x_{i},y_{i}\right)\right\}}_{i=1}^{N}\right)$$

where

(6) $$\begin{array}{c} \text{CSA}\left({\left\{\left(x_{i},y_{i}\right)\right\}}_{i=1}^{N}\right)=\text{MHSA}\left(\text{Concat}\left({\left\{f\left(x_{i},y_{i}\right)\right\}}_{i=1}^{N},\text{CPE}\left({\left\{\left(x_{i},y_{i}\right)\right\}}_{i=1}^{N}\right)\right)\right) \end{array}$$

where *f*(*x*, *y*) represents the feature of the point extracted from $F^{\prime }$ (obtained by applying several convolution layers to F), and $\text{CPE}=\{\left(x_{i}^{\prime },y_{i}^{\prime }\right)|i=1,2,\cdots ,N\}$, where $\left(x_{i}^{\prime },y_{i}^{\prime }\right)$ can be calculated as follows:

(7) $$x_{i}^{\prime }=\frac{x_{i}-x_{min}}{x_{max}-x_{min}},\;y_{i}^{\prime }=\frac{y_{i}-y_{min}}{y_{max}-y_{min}}$$

where (*x*_*min*_, *y*_*min*_) and (*x*_*max*_, *y*_*max*_) indicate the minimum and maximum values determined over all vertices of the contour, respectively. The initial contour was then refined to {(*x*_*i*_ + Δ*x*_*i*_, *y*_*i*_ + Δ*y*_*i*_)∣*i* = 1, 2, …, *N*}. The scaled dot‐product attention computation method was adopted in the MHSA block,^[^ ^25^ ^]^ and it can be formulated as shown below:

(8) $$\begin{array}{cc} \text{MHSA}(P) & =P⊕\left(\text{ReLU}\left(P⊕\text{Attention}\left(P\right)\right)W^{z_{1}}\right)W^{z_{2}}\\ & \;\;\text{where}\;W^{z_{1}}\in R^{d\times 4d}\;\text{and}\;W^{z_{2}}\in R^{4d\times d}\\ \text{Attention}(P) & =\text{Concat}\left({\text{head}}_{1},\cdots ,{\text{head}}_{h}\right)W^{O}\\ & \;\;\text{where}\;W^{O}\in R^{d\times d}\\ {\text{head}}_{h} & =\text{softmax}\left(\frac{Q_{h}K_{h}^{⊤}}{\sqrt{d_{h}}}\right)V_{h} \end{array}$$

where $P\in R^{N\times d}$ represents the input sequence and *W* denotes the matrix of learnable parameters. The vectors *Q*_*h*_, *K*_*h*_, *V*_*h*_ were generated via linear transformations: $Q_{h}=PW_{h}^{Q}$, $K_{h}=PW_{h}^{K}$, $V_{h}=PW_{h}^{V}$, where $W_{h}^{Q},W_{h}^{K},W_{h}^{V}\in R^{d\times d_{k}}$ and *d*_*k*_ = *d*/*H*. The index *h* refers to the heads, with *H* heads in total. In this experiment, *N* was set to 128, *d* was set to 254+2, and *H* was set to 8, with 2 CSA blocks employed.

### CIEN Implementation

To supervise the predicted contours, a soft assignment strategy was employed; this strategy does not require the exact pairing of the predicted points and GT points but instead treats them as samples acquired from two probability distributions, minimizing the distance between them. To implement this strategy, OT theory was used to match the two distributions. OT refers to the optimal cost of transforming one probability distribution into another, providing a differentiable process that facilitates the training procedure of the CIEN. The implementation scheme that applies OT theory to the supervision task was detailed here.

**Encoding Measures**: Let $\Psi ={\left\{\psi_{i}|\psi_{i}\in R^{2}\right\}}_{i=1}^{N}$ and $\Phi ={\left\{\phi_{j}|\phi_{j}\in R^{2}\right\}}_{j=1}^{N}$ denote the sets of predicted and GT points in a 2D vector space, respectively. Let (α, β) be two discrete measures with weights of (*a*, *b*) and locations at (Ψ, Φ), which are defined as follows:
(9) $$\alpha =\sum_{i=1}^{N}a_{i}\delta_{\psi_{i}},\;\beta =\sum_{j=1}^{N}b_{j}\delta_{\phi_{j}}$$
  where δ_*x*_ denotes the Dirac delta function at position *x*; $a,b\in R_{+}^{N}$ with $\sum_{i=1}^{N}a_{i}=1$ and $\sum_{j=1}^{N}b_{j}=1$.
**OT Formulation**: Let $c:\Psi \times \Phi \mapsto R_{+}$ be the function for calculating the cost of moving a point from Ψ to a point in Φ, and let *C* be the corresponding *N* × *N* cost matrix, where *C*_*ij*_ = *c*(ψ_*i*_, ϕ_*j*_),). The OT problem aims to find an optimal map {ψ_1_, …, ψ_*i*_} → {ϕ_1_, …, ϕ_*j*_} that minimizes the overall cost. Under Kantorovich relaxation,^[^ ^28^ ^]^ the possible maps Γ can be defined as follows:
(10) $$\Gamma \left(a,b\right)\overset{\text{def.}}{=}\left\{\gamma \in R_{+}^{N\times N}:\gamma 1_{N}=a\;\text{and}\;\gamma^{⊤}1_{N}=b\right\}$$
  where γ is the coupling matrix that represents the transportation plan. The OT loss for seeking the minimal transportation cost incurred when transferring α to β can be formulated as shown below:^[^ ^39^ ^]^
(11) $$\text{OT}\left(\alpha ,\beta \right)=\min_{\gamma \in \Gamma (a,b)}\left\langle C,\gamma \right\rangle =\sum_{i,j}C_{i,j}\gamma_{i,j}$$
**Regularization and Debiasing**: Since solving the original OT problem directly was complex, an approximate solution was typically obtained through a dual formulation with a regularization parameter ε:^[^ ^40^ ^]^
(12) $$\begin{array}{c} {\text{OT}}_{\varepsilon }\left(\alpha ,\beta \right)=\max_{f,g\in R^{N}}\left\langle \alpha ,f\right\rangle +\left\langle \beta ,g\right\rangle -\varepsilon \left\langle \alpha ⊗\beta ,\exp\left(\frac{1}{\varepsilon }\left(f⊕g-C\right)\right)-1\right\rangle \end{array}$$
  where (*f*, *g*) is the optimal dual pair for solving the equation. Owing to the regularization penalty, OT_ε_(α, β) does not vanish when α = β. Thus, the debiased Sinkhorn divergence was adopted as the final loss:^[^ ^41^ ^]^
(13) $$L_{\text{OT}}\overset{\text{def.}}{=}{\text{OT}}_{\varepsilon }\left(\alpha ,\beta \right)-\frac{1}{2}OT_{\varepsilon }\left(\alpha ,\alpha \right)-\frac{1}{2}{\text{OT}}_{\varepsilon }\left(\beta ,\beta \right)$$
**Solving OT and Gradients**: An efficient computational scheme for the Sinkhorn algorithm was provided by Ref. [42] through symmetrization and debiasing, allowing ε to decay from a large initial estimate to a small target value across numerous iterations. The gradients can then be computed as follows:^[^ ^42^ ^]^
(14) $$\begin{array}{cc} \frac{1}{a_{i}}\nabla_{\psi_{i}}L_{\text{OT}} & =\frac{\sum_{j=1}^{N}\exp\left(\frac{1}{\varepsilon }\left[g_{j}^{\alpha \to \beta }-c\left(\psi_{i},\phi_{j}\right)\right]\right)\nabla_{\psi_{i}}c\left(\psi_{i},\phi_{j}\right)}{\sum_{j=1}^{N}\exp\left(\frac{1}{\varepsilon }\left[g_{j}^{\alpha \to \beta }-c\left(\psi_{i},\phi_{j}\right)\right]\right)}\\ & -\frac{\sum_{k=1}^{N}\exp\left(\frac{1}{\varepsilon }\left[f_{k}^{\alpha \to \alpha }-c\left(\psi_{i},\psi_{k}\right)\right]\right)\nabla_{\psi_{i}}c\left(\psi_{i},\psi_{k}\right)}{\sum_{k=1}^{N}\exp\left(\frac{1}{\varepsilon }\left[f_{k}^{\alpha \to \alpha }-c\left(\psi_{i},\psi_{k}\right)\right]\right)} \end{array}$$
  where *f* ^α → α^ and *g* ^α → β^ are the dual potentials in OT_ε_(α, α) and OT_ε_(α, β), respectively.

In this experiment, the points in Ψ and Φ were considered equally important for contour refinement purposes, so *a*_*i*_ and *b*_*j*_ were set to $\frac{1}{N}$. The cost function *c*(ϕ_*i*_, ψ_*j*_) employed the Euclidean distance measure, which was appropriate for coordinate attributes: *c*(ϕ_*i*_, ψ_*j*_) = ‖ϕ_*i*_ − ψ_*j*_‖_2_. The target ε and the scaling ratio *q* used in Feydy's computation were set to 0.05 and 0.5, respectively.

For the centre heatmap predictions, the focal loss^[^ ^43^ ^]^ was employed as the loss function (denoted as $L_{\text{focal}}$). The overall loss function was defined as follows:

(15) $$L=L_{\text{focal}}+0.5L_{\text{OT}}^{init}+0.5L_{\text{OT}}^{{\text{CSA}}_{1}}+L_{\text{OT}}^{{\text{CSA}}_{2}}$$

In this study, *N* was empirically set to 128, which effectively covered most pig shapes. Given the straightforward nature of the task, which involved performing instance segmentation within a single category, and the simplicity of the pig farm environment, stratified sampling was employed to obtain a mini dataset from LISAP at a rate of 1% for conducting comparative research. During training, random horizontal flipping, rotation, scaling, and color augmentation were applied as data augmentation methods. The CIEN model was trained for 300 epochs via the adaptive moment estimation (Adam) optimizer,^[^ ^44^ ^]^ with an initial learning rate of 0.0001. This learning rate was systematically reduced by a factor of two at milestones of 50, 100, 150, 200, 250, and 270 epochs to facilitate efficient convergence. The training process was performed on 4 RTX 3090 GPUs with a batch size of 24, ensuring the optimal utilization of the available computational resources. More training details can be found in Table S6 (Supporting Information).

### The Workflow of LWEN

Let $X=\left\{\left(x_{i},y_{i}\right)|i=1,2,\cdots ,N\right\},X\in R^{N\times 2}$ denote a set of points for one object, which might be ordered or unordered. W represents a learnable parameter matrix. The workflow of the LWEN, as shown in Figure 8, is described in the following steps.

i. The input sequence X is normalized relative to the image *I*, where $I\in {\text{Mat}}_{H\times W}\left(R\right)$ and itself:
(16) $$X^{norm}=\text{Concat}\left(\text{ImgNorm}\left(I,X\right),\text{BoxNorm}\left(X\right)\right)$$
  where
(17) $$\text{ImgNorm}\left(I,X\right)={\left\{\left(\frac{x_{i}-W}{W},\frac{y_{i}-H}{H}\right)\right\}}_{i=1}^{N}$$
  and BoxNorm(X) is computed using Equation (7) and regarded as the positional embedding.
ii. The normalized sequence $X^{norm}\in R^{N\times 4}$ is projected to a high‐dimensional feature space via linear projection (*lp*):
(18) $$X^{lp}=X^{norm}W^{lp},\;W^{lp}\in R^{4\times d_{hide}}$$
iii. The features are aggregated via the MHSA mechanism, as expressed in Equation (8):
(19) $$X^{m}=\text{MHSA}\left(X^{lp}\right),\;X^{lp},X^{lp}\in R^{N\times d_{hide}}$$
iv. The average values along the feature channels were computed to obtain a feature vector: $X^{f}\in R^{d_{hide}}$.
v. The predicted liveweight LW was regressed from $X^{f}$ through a fully connected layer:
(20) $$\begin{array}{c} \text{LW}=\left(\text{LeakyReLU}\left(X^{f}W^{fc_{1}}\right)\right)W^{fc_{2}},\;W^{fc_{1}}\in R^{d_{hide}\times d_{fc}},\;W^{fc_{2}}\in R^{d_{fc}\times 1} \end{array}$$

In this study, *N* was set to 128, *d*_*hide*_ was set to 512, *d*_*fc*_ was set to 128, and 8 heads were used within the MHSA mechanism. The smooth L1 loss^[^ ^45^ ^]^ was utilized to supervise the predictions. The LWEN was trained via the Adam optimizer with a learning rate of 0.001 and a weight decay rate of 0.0005. A warm‐up strategy with a factor of 0.1 was applied for the first 10 epochs. The LWEN was trained for 400 epochs, and its learning rate was decreased by a factor of 10 at epochs 80, 160, 240, and 320. The input sequence was uniformly sampled from the GT during training and predicted by the CIEN during testing. Random horizontal flipping and rotation were used as training data augmentation techniques. Additionally, two methods were employed for training augmentation purposes: 1) obtaining *N* points from the 4 × *N* GT points through uniform sampling and 2) adding random noise to the input sequence. The LWEN algorithm was implemented on 4 RTX 3090 GPUs with a batch size of 32. More training details can be found in Table S6 (Supporting Information). The hardware configuration details for this study can be found in Table S7 (Supporting Information).

### Statistical Analysis

In this study, IBM SPSS Statistics for Windows, version 26, was used for statistical analysis (Section 2.4.2). The data for statistical analysis consisted of relative error distributions for each day, which contains a total of 21 groups each with the sample size of 13. And the above data had not undergone any pre‐processing. Prior to statistical comparisons, the data were tested for normality using the Shapiro‐Wilk test and for homogeneity of variance using the Levene test. For statistical comparisons, a one‐way analysis of variance (ANOVA) was conducted, followed by the least significant difference (LSD) post‐hoc test to assess significant differences among groups. Intergroup differences were considered statistically significant when p < 0.05.

## Conflict of Interest

The authors declare no conflict of interest.

## Supporting information

Supporting Information

## Data Availability

The dataset utilized in this study are publicly accessible, ensuring transparency and reproducibility of the findings. In keeping with best practices in open science, we encourage readers to explore, reuse, and extend these datasets for their own research purposes. The dataset and source code presented in this manuscript are available at https://github.com/TXiang‐lab/CIEN‐LWEN.
