# Supplementary material for: A Novel Dual‐Network Approach for Real‐Time Liveweight Estimation in Precision Livestock Management
Source: Adv Sci (Weinh). 2025 Apr 26;12(22):2417682. doi: 10.1002/advs.202417682 (PMC12165045; doi:10.1002/advs.202417682)
Supplement: Supplementary file 1 — Supporting Information [file ADVS-12-2417682-s001.pdf]

ADVANCED SCIENCE
Open Access

Supporting Information

for *Adv. Sci.*, DOI 10.1002/advs.202417682

A Novel Dual-Network Approach for Real-Time Liveweight Estimation in Precision Livestock Management

Ximing Dong, Caiming Zhang, Peiyuan Wang, Dexuan Chen, Gang Jun Tu, Shuhong Zhao* and Tao Xiang**

A Novel Dual-Network Approach for Real-Time Liveweight Estimation in Precision Livestock Management – **Supporting Information**

Ximing Dong Caiming Zhang Peiyuan Wang Dexuan Chen Gang Jun Tu Shuhong Zhao* Tao Xiang**

X. Dong, C. Zhang, P. Wang, D. Chen, G. J. Tu, S. Zhao, T. Xiang
Key Laboratory of Agricultural Animal Genetics, Breeding and Reproduction of Ministry of Education & Key Laboratory of Swine Genetics and Breeding of Ministry of Agriculture, Huazhong Agricultural University, Wuhan, China
Email Address:GangJunTu@mail.hzau.edu.cn, shzhao@mail.hzau.edu.cn, Tao.Xiang@mail.hzau.edu.cn

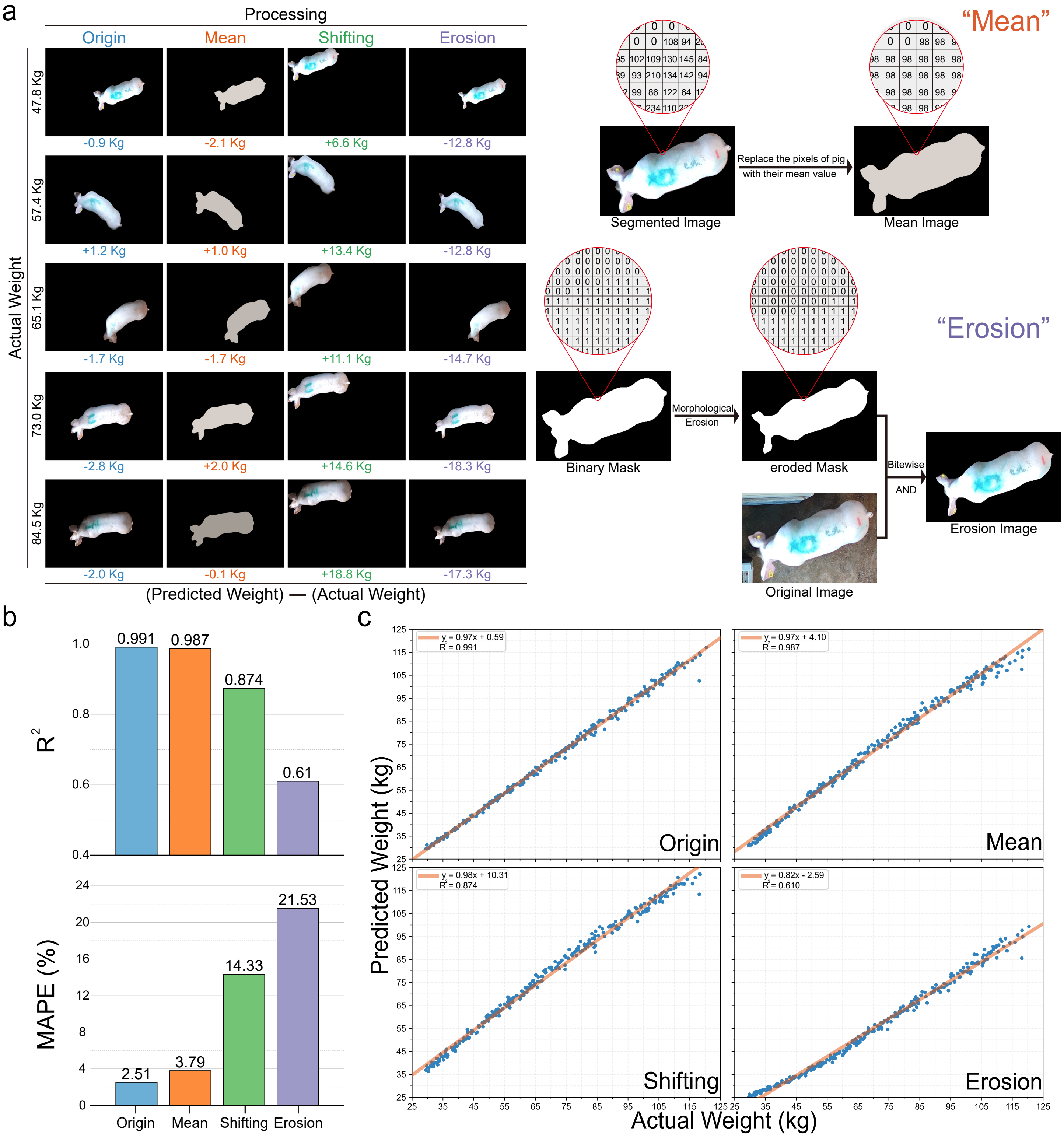

Figure S1: Impact of texture, positional, and geometric modifications on pig liveweight prediction accuracy Using a MobileNet trained on segmented images. "Mean" indicates that all pixels were replaced with their mean value, "Shifting" refers to repositioning the pigs to the top-left corner, and "Erosion" denotes the application of morphological erosion to the pig image. (a) Visualization of the three test setups: texture removal ("Mean"), positional shift ("Shifting"), and morphological erosion ("Erosion"). (b) Prediction performance under each test setup. (c) Regression analysis illustrating the effects of different modifications on liveweight estimation.

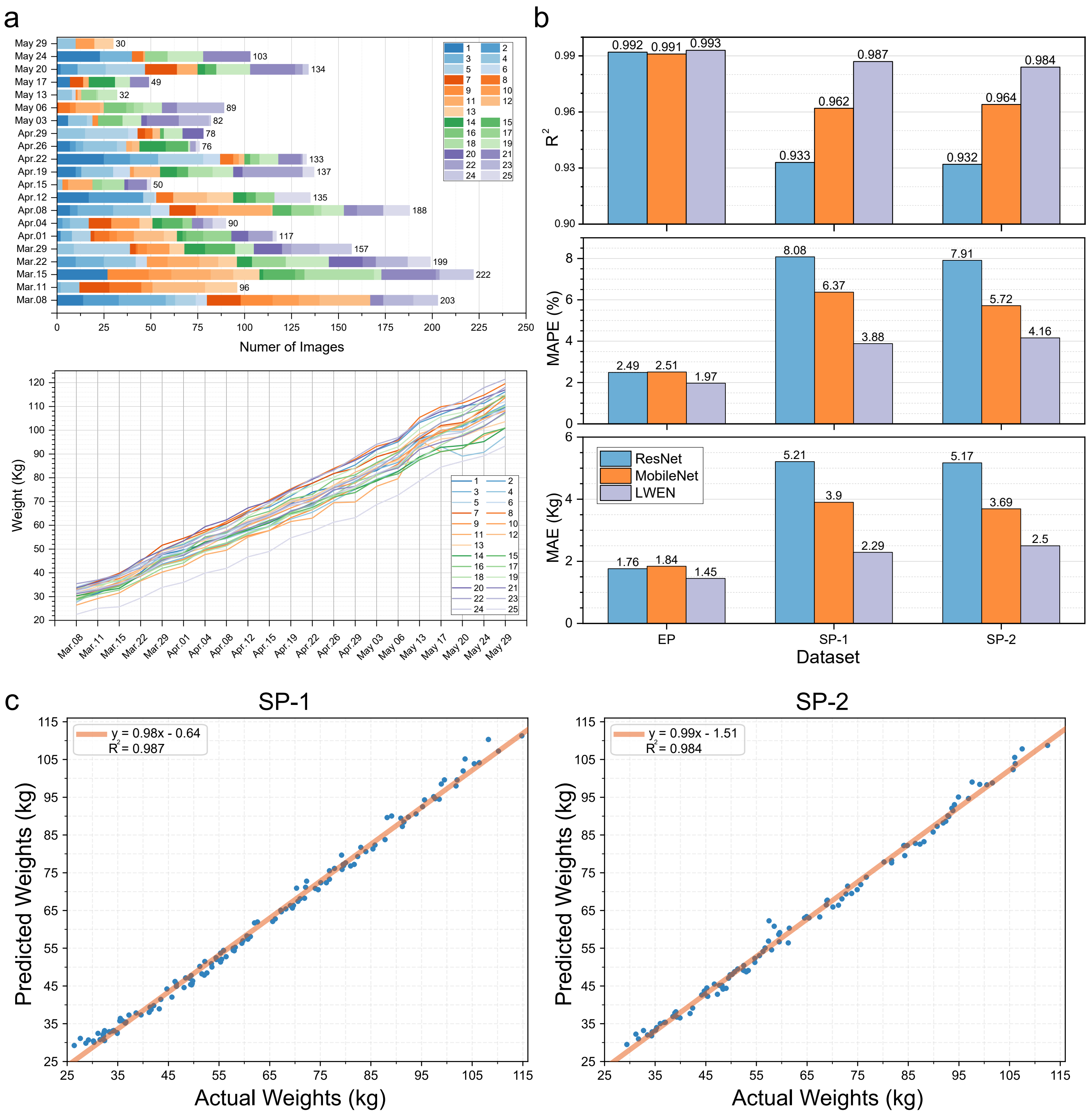

Figure S2: Cross-pen evaluation of the CIEN-LWEN framework in supplementary pens (SP-1 and SP-2). The framework are trained exclusively on the training set of experimental pen(EP) of LISAP dataset. (a) Image counts (above) and weight distributions(below) in SP-1 and SP-2. The first 13 pigs are in SP-1, while the 14th to 25th pigs are in SP-2. (b) Performance comparison of framework and CNN-based methods in SP-1, SP-2, and EP. (c) Regression analysis of actual and predicted weights in SP-1 and SP-2 using the proposed framework.

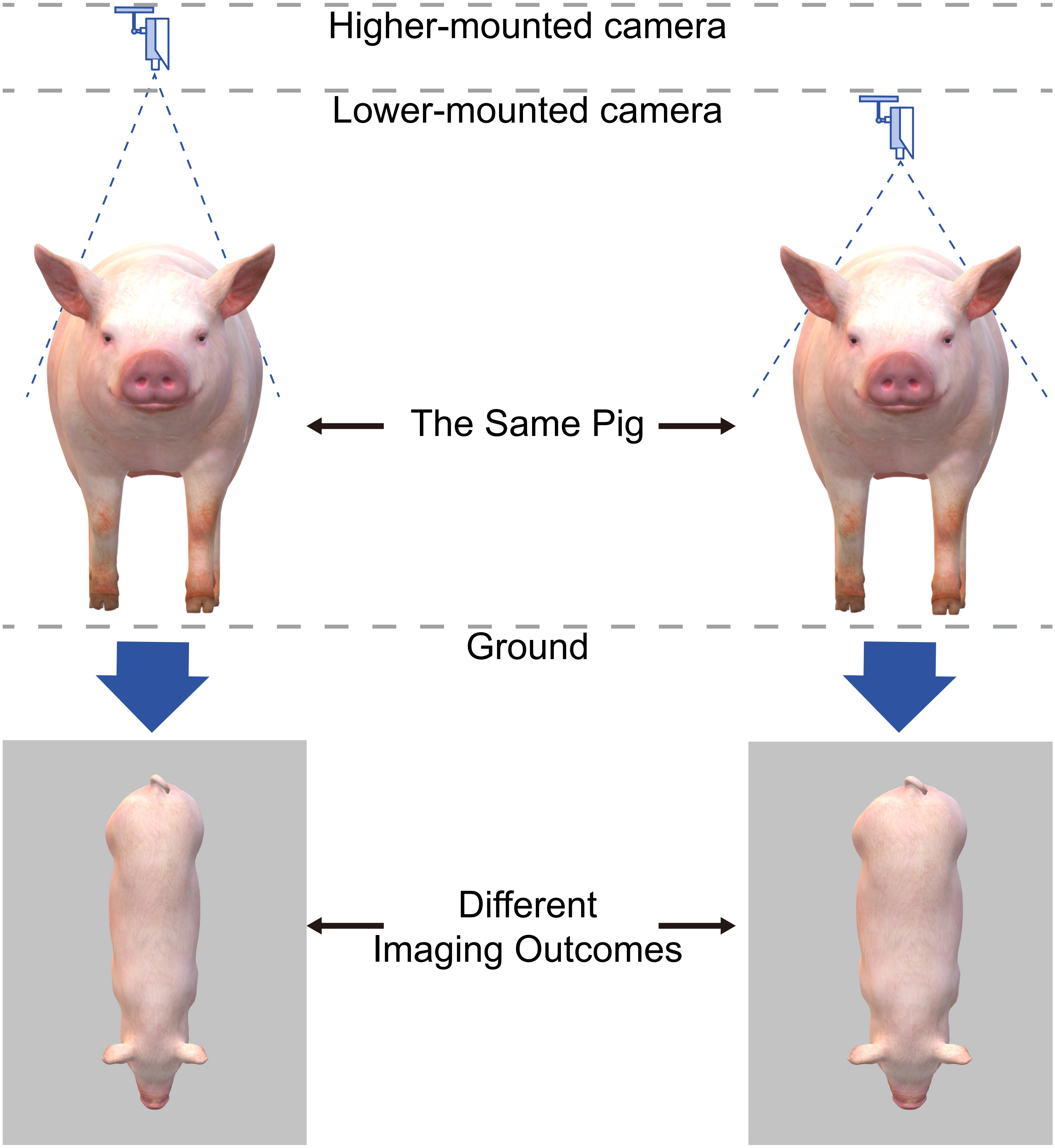

Figure S3: Impact of camera installation parameters on pig imaging outcomes. Lower-mounted cameras tend to produce larger swine dimensions, while higher-mounted cameras capture smaller sizes.

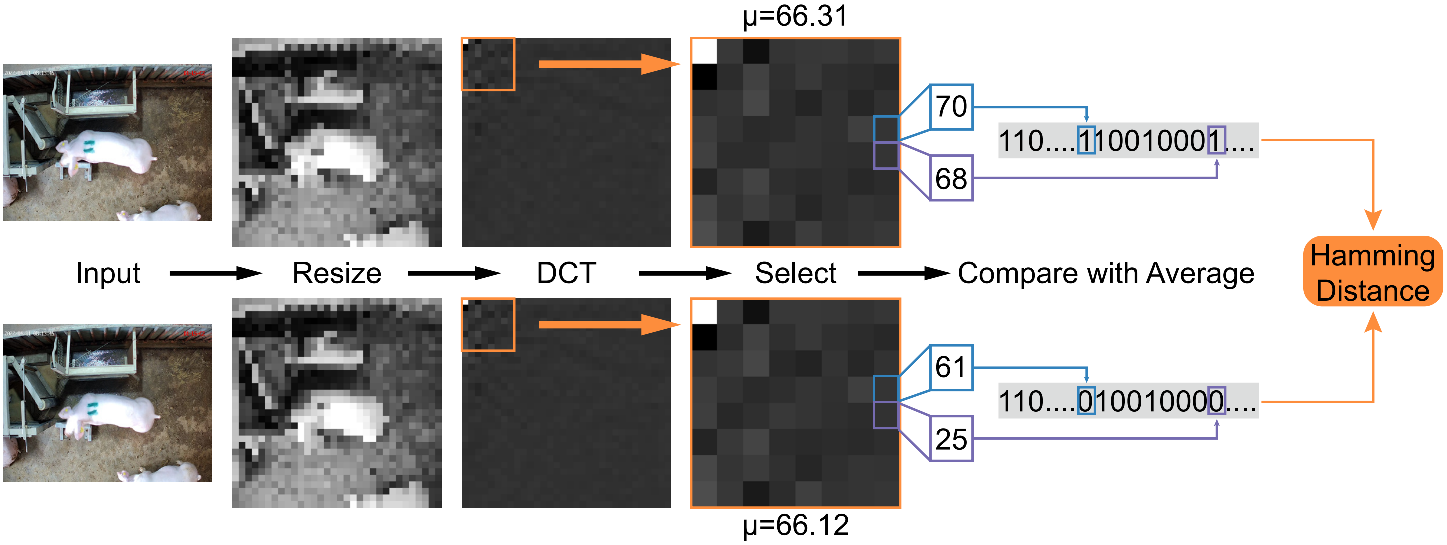

Figure S4: The perceptual hash used for filtering similar images. Two images will be considered similar if their hamming distance is less than or equal to 15 in this study.

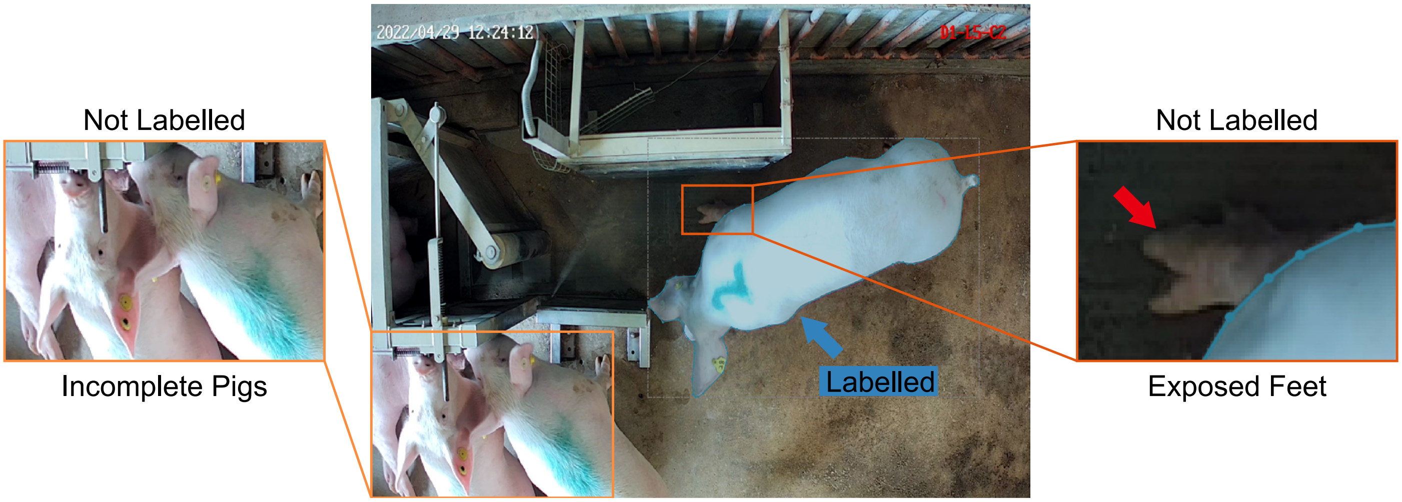

Figure S5: The example of annotation details of LISAP in this study. The pigs that have whole contour in the image except the area of exposed feet or legs are labelled.

Table S1: The recorded liveweights of each pig each day.

ID \ Date	3/8/22	3/11/22	3/15/22	3/22/22	3/29/22	4/1/22	4/4/22	4/8/22	4/12/22	4/15/22	4/19/22	4/22/22	4/26/22	4/29/22	5/3/22	5/6/22	5/13/22	5/17/22	5/20/22	5/24/22	5/29/22
1	33	35	39.6	44.4	51.2	54.9	57.6	62.5	64.2	68.3	72.1	74.9	81.7	84.1	88.7	89.9	99	103.2	103.5	108.2	111.6
2	33.7	36.4	38.6	43.4	48.6	52.9	57	60	63.9	67.8	72.7	76.7	80.9	83.4	89.3	92.9	99.2	103.9	106.9	112.3	118.4
3	29.5	29.8	31.6	36.5	42.4	42.9	48	48.1	53.2	56.5	61.2	65.6	71.6	74.6	81.1	83.1	91.7	96.3	97.9	102.2	105.3
4	33.8	36.3	39	46.2	52.1	53.7	56	60.3	63.1	67	70.4	78.3	82	84.3	89.2	92.9	100.3	106.6	109.7	115.9	118.1
5	33.4	35.8	37.8	43	49.6	52.9	56.8	60.6	65.1	68.4	71.4	75.9	78.7	82.4	86.7	89.7	95.1	100.8	102.7	106	111.2
6	31.4	33.3	35.8	40.3	44.1	47.8	51	53.6	58	61.7	64.2	67.1	71.8	77.1	81	83.3	92.1	97.4	100.6	103.8	110.6
7	30.4	32.3	34.5	40.3	46.7	48.3	50.5	54.4	57.4	62.4	65.8	70.1	73.6	77.1	82.2	85.4	91.1	96.2	98.3	101.7	105.8
8	34.2	36.1	38.6	42.9	49.1	52.5	56.2	58.8	63.4	65.9	69.5	74.6	78.5	79.6	85.8	90.3	95.2	99.2	101.8	105.4	108.8
9	31.8	35.2	38.9	43.5	49.8	52.7	57.4	61.2	64.7	70.2	74.6	77.1	79	84.4	83.7	89.5	98.1	99.7	106.2	107.4	110
10	32	32.8	33.9	38.4	41.8	44.3	46.5	49.8	53.2	56.1	62.2	66.6	72.4	75.7	80.2	84	91.2	95.3	100.5	105.5	110
11	33.3	35.8	38.2	44.7	50.2	52.1	56.5	60.6	63.2	67.3	73	79.6	83	87	93.8	95.9	105.4	108.9	112.8	116.8	120.4
12	31	32.9	35	39.1	45.8	46.7	50.7	51.8	55.7	59	64.9	68.3	74.5	78.7	84.5	87.4	94.9	101.3	103.9	110.4	114.2
13	30.2	33.3	36.6	41.5	49.3	52.4	56.7	60.2	64.5	68.2	72.4	77.8	82.6	84.4	91	94.3	101.4	105.5	107.8	113.3	118.2

Table S2: Pseudocode for CIEN - Contour Information Extraction Network

**Input:** Image I
**1. Feature Extraction:**<br>Use MobileNetV2 to extract feature maps F from input image I.<br>Aggregate multi-scale features using Iterative Deep Aggregation (IDA) to obtain F_{agg}.
**2. Contour Initialization:**<br>Initialize coarse contour $\mathcal{C}_{init}$ from the center point.<br>$\mathcal{C}_{init} \leftarrow$ regress contour from center point to contour vertices.
**3. Contour Refinement:** Contour Self-Attention (CSA)<br>Compute Contour Positional Embedding (CPE) for points in $\mathcal{C}_{init}$.<br>Apply Multi-Head Self-Attention (MHSA) to model long-range dependencies between points.<br>Fuse global context information with positional awareness.<br>Predict offset $\Delta\mathcal{C}$ for each contour point.
**4. Update Contour:** $\mathcal{C} \leftarrow \mathcal{C}_{init} + \Delta\mathcal{C}$.
**5. Supervision and Optimization:**<br>**5.1 HardAssign Strategy**<br>Minimize distance between predicted points and ground-truth (GT) points using Kuhn-Munkres (KM) algorithm.<br>$\text{Loss}_{\text{HardAssign}} \leftarrow \sum_i \|\mathcal{C}_{pred}[i] - \mathcal{C}_{GT}[i]\|^2$.<br>**5.2 SoftAssign Strategy**<br>Use Optimal Transport (OT) theory to align predicted contour distribution with GT distribution.<br>$\text{Loss}_{\text{SoftAssign}} \leftarrow \text{OT}(\mathcal{C}_{pred}, \mathcal{C}_{GT})$.
**Final Output:** Refined contour $\mathcal{C}$

Table S3: Comparison among the instance segmentation results produced by different contour evolution methods.

Evolution Block	Smooth L1 Loss			OT Loss			Params↓	FPS↓
	AP↑	AP^{50} ↑	AP^{75} ↑	AP↑	AP^{50} ↑	AP^{75} ↑		
Contour Self-Attention	99.00	**99.91**	**99.91**	**99.56**	**99.92**	**99.92**	**3.8M**	**53.7**
Global Deformation	97.96	98.98	98.98	98.55	98.98	98.98	36.0M	48.0
Circular Convolution	**99.07**	99.85	99.85	99.33	99.84	99.84	42.0M	42.8

Table S4: The pseudocode for the proposed LWEN

Input:
Contour coordinates of pigs, Image, Bounding box

1. Normalize contour coordinates:
Normalize coordinates relative to the image and bounding box

2. Project normalized coordinates:
Apply linear projection to map coordinates to higher-dimensional space

3. Extract features:
Apply Multi-Head Self-Attention (MHSA)
for each head in MHSA:
Capture relationships and aggregate features
end for

4. Liveweight Estimation:
Average features across channels
Apply fully connected layer for final regression

5. Output:
Predict live weight based on processed features

Table S5: The liveweight prediction results obtained by enhancing the width and depth of LWEN.

Network	MAE↓	MSE↓	MAPE↓	R^2 ↑	Params↓	FPS↑
LWEN	1.45	4.45	1.97	0.993	3.2M	1131.6
LWEN-Wide	1.39	3.98	1.93	0.994	12.7M	1088.6
LWEN-Deep	1.36	3.93	1.84	0.994	17.0M	207.6

Table S6: The hyperparameters for training CIEN and LWEN

Hyperparameters	CIEN	LWEN
epochs	300	400
learning rate	0.0001	0.001
weight decay	0.0005	0.0005
milestones[a]	[50, 100, 150, 200, 250, 270]	[80, 160, 240, 320]
gamma[a]	0.5	0.1
warm up[b]	\	epochs:10, factor:0.1
optimizer	Adam	Adam
batch size per GPU	24	32
python version	3.7.16	3.7.16
pytorch version	1.8.1	1.8.1
CUDA version	11.1	11.1

[a] This indicates that when the training process(epoch) reaches a milestone, the current learning rate will be multiplied by the gamma.
[b] This indicates that the network will begin training by multiplying the learning rate by the factor, and then increase it linearly after each epoch until it reaches the set value at the specified epoch.

Table S7: Hardware configuration details for this study

Componet	Specification
CPU	2 × Intel Xeon Platinum 8358, 32 cores, 2.6 GHz
GPU	4 × NVIDIA RTX 3090, 24GB, 10496 CUDA cores
RAM	128 × 128 GB DDR4, 3200 MHz
Storage	8 × 20 TB HDD, 7200 rpm
Operating System	Ubuntu 20.04.4 LTS
